# Microwave-assisted design of nanoporous graphene membrane for ultrafast and switchable organic solvent nanofiltration

**DOI:** 10.1038/s41467-023-36524-x

**Published:** 2023-02-17

**Authors:** Junhyeok Kang, Yeongnam Ko, Jeong Pil Kim, Ju Yeon Kim, Jiwon Kim, Ohchan Kwon, Ki Chul Kim, Dae Woo Kim

**Affiliations:** 1 Department of Chemical and Biomolecular Engineering, Yonsei University, Yonsei-ro 50, Seodaemun-gu, Seoul (03722) Republic of Korea; 2 Department of Chemical Engineering, Konkuk University, Seoul, 05029 Republic of Korea

**Keywords:** Synthesis of graphene, Synthesis and processing

## Abstract

Layered two-dimensional materials can potentially be utilized for organic solvent nanofiltration (OSN) membrane fabrication owing to their precise molecular sieving by the interlayer structure and excellent stability in harsh conditions. Nevertheless, the extensive tortuosity of nanochannels and bulky solvent molecules impede rapid permeability. Herein, nanoporous graphene (NG) with a high density of *sp*^2^ carbon domain was synthesized via sequential thermal pore activation of graphene oxide (GO) and microwave-assisted reduction. Due to the smooth *sp*^2^ carbon domain surfaces and dense nanopores, the microwave-treated nanoporous graphene membrane exhibited ultrafast organic solvent permeance (e.g., IPA: 2278 LMH/bar) with excellent stability under practical cross-flow conditions. Furthermore, the membrane molecular weight cut-off (MWCO) is switchable from 500 Da size of molecule to sub-nanometer-size molecules depending on the solvent type, and this switching occurs spontaneously with solvent change. These properties indicate feasibility of multiple (both binary and ternary) organic mixture separation using a single membrane. The nanochannel structure effect on solvent transport is also investigated using computation calculations.

## Abstract (web summary)

Layered 2D materials can be used for organic solvent nanofiltration (OSN) membrane fabrication due to precise molecular sieving by the interlayer structure and stability in harsh conditions. Here authors synthesise sp^2^-enriched nanoporous graphene by microwave treatment and demonstrate its excellent OSN performance.

## Introduction

Chemical purification is a crucial process in various industries, such as petrochemicals and pharmaceuticals, to purify valuable products and recycle solvents or catalysts^1^. Conventional purification processes based on distillation and crystallization are costly and inapplicable for heat-sensitive products^2^. Alternatively, organic solvent nanofiltration (OSN) membranes capable of separating molecules between 100–2000 Da in size are promising owing to their environmentally friendly operation, high energy efficiency, and integrated system^3,4^. Specifically, when the membrane has switchable molecular separation properties by external stimuli or chemical conditions, the OSN-based separation process can be further integrated and made cost-effective because most practical chemical purification processes aim to separate multiple mixed molecules. As a proof of concept, few membranes with switchable molecular separation properties have been demonstrated using covalent organic networks (CON), porous organic cages (POC), metal-organic frameworks (MOF), and functionalized graphene^5–8^.

Graphene and its derivatives are the most widely utilized two-dimensional (2D) materials for membrane fabrication, which has been widely applied to separation applications such as gas separation, water treatment, organic solvent purification, pervaporation, and nanofiltration^9–15^. Particularly, graphene oxide (GO) is effective for membrane fabrication because large-scale fabrication is feasible via practical continuous coating methods (bar or slot-die coating) and its narrow interlayer spacing enables precise molecular separation^11,16,17^. However, narrow interlayer spacing and lengthy diffusion pathways typically result in limited organic solvent flow^18^. Therefore, various approaches have been reported to modify nanochannel structure, such as pore generation, flake size control, and hybridization with other nanomaterials, which can decrease solvent diffusion length or increase nanochannel size^18–20^. While these methods can produce OSN membrane with rapid solvent permeation, switchable molecular separation phenomena have been rarely reported and the majority of membranes have been utilized to separate single or binary mixtures^18,21^.

Herein, nanoporous graphene with rich sp^2^ carbon domains were prepared for OSN membrane fabrication. Dense nanopores on the basal plane of graphene were activated by spontaneous thermal annealing of GO. Subsequently, microwave treatment was conducted to increase the proportion of sp^2^ carbon domains by transforming defective sp^3^ carbons at elevated local temperatures. Graphitic structural changes owing to thermal activation and microwave treatment were systemically investigated. The membrane OSN performances were investigated using a dead-end and cross-flow system. Furthermore, molecular separation properties of microwave-treated nanoporous graphene (MNG) membrane were demonstrated to be switchable in response to the solvents employed to separate multiple mixtures of subnanometer-sized organic molecules through repetitive solvent exchange and filtration using a single membrane.

## Results

### Fabrication of microwave-treated NG membrane

**Fig. 1:**
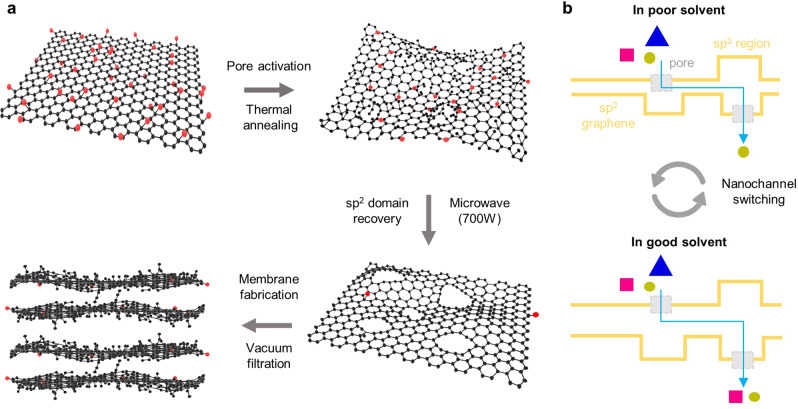
Microwave-assisted design of nanoporous graphene membrane. **a** Preparation of sp^2^ carbon enriched nanoporous multilayer graphene membrane enabled by microwave-assisted reduction. **b** Membrane working principle for switchable molecular separation in response to solvent types.

Figure 1a depicts the overall fabrication procedure for MNG membranes. First, to prepare nanoporous graphene (NG), the as-prepared GO powder was thermally annealed at 200 °C for 5 min under ambient conditions, which activates numerous nanopores on the graphene surface by decomposing oxygen-containing groups^22^. Thermal annealing is effective at producing dense nanopores; however, oxygen group decomposition frequently results in amorphous sp^3^ carbon formation. Because the sp^3^ carbon structure may impede solvent molecule transport owing to steric hindrance^23,24^, NG was treated with microwave to recover sp^2^ domains on the graphene surface^25^. Voiry et al. reported that high-quality graphene composed with a high proportion of sp^2^ carbons can be prepared via treating reduced graphene oxide with microwave^25^, while microwave treatment effects on nanoporous graphene have been not investigated. In addition, the microwave treatment can generate denser nanopores during sp^3^ carbon rearrangement or decomposition, further enhancing solvent permeation. Notably, previous chemical and thermal reduction methods utilized for graphene membranes solely remove oxygen-containing groups with partial recovery of sp^2^ domains because recrystallization of amorphous carbons require extremely high temperatures above 2000 °C^25–27^. Furthermore, as thermally activated nanoporous graphene is extremely defective, conventional thermal and chemical reduction methods are inapplicable. In contrast, the microwave treatment is effective at recrystallizing the sp^3^ carbon structure by increasing the local temperature of graphene owing to its explosive carrier scattering under microwave irradiation^28^.

The prepared MNG powder was dispersed in 1-methyl-2-pyrrolidone (NMP) via sonication. After removing large particles and unexfoliated flakes by centrifugation, the MNG solution was vacuum filtered on nylon support. Figure 1b illustrates the nanochannel structures of MNG membrane. The dense nanopore can effectively provide additional flow channels and filter large molecules. Owing to sp^2^ domain recovery after microwave treatment, the MNG membrane can possess a narrower interlayer spacing than the NG membrane, and the remaining sp^3^ carbons serve as spacers to support stacked graphene sheets. In addition, the nanochannel size of the MNG membrane is spontaneously variable based on the swelling degree of organic solvents within the MNG layer, resulting in switchable molecular separation phenomena, which is subsequently described in detail.

**Fig. 2:**
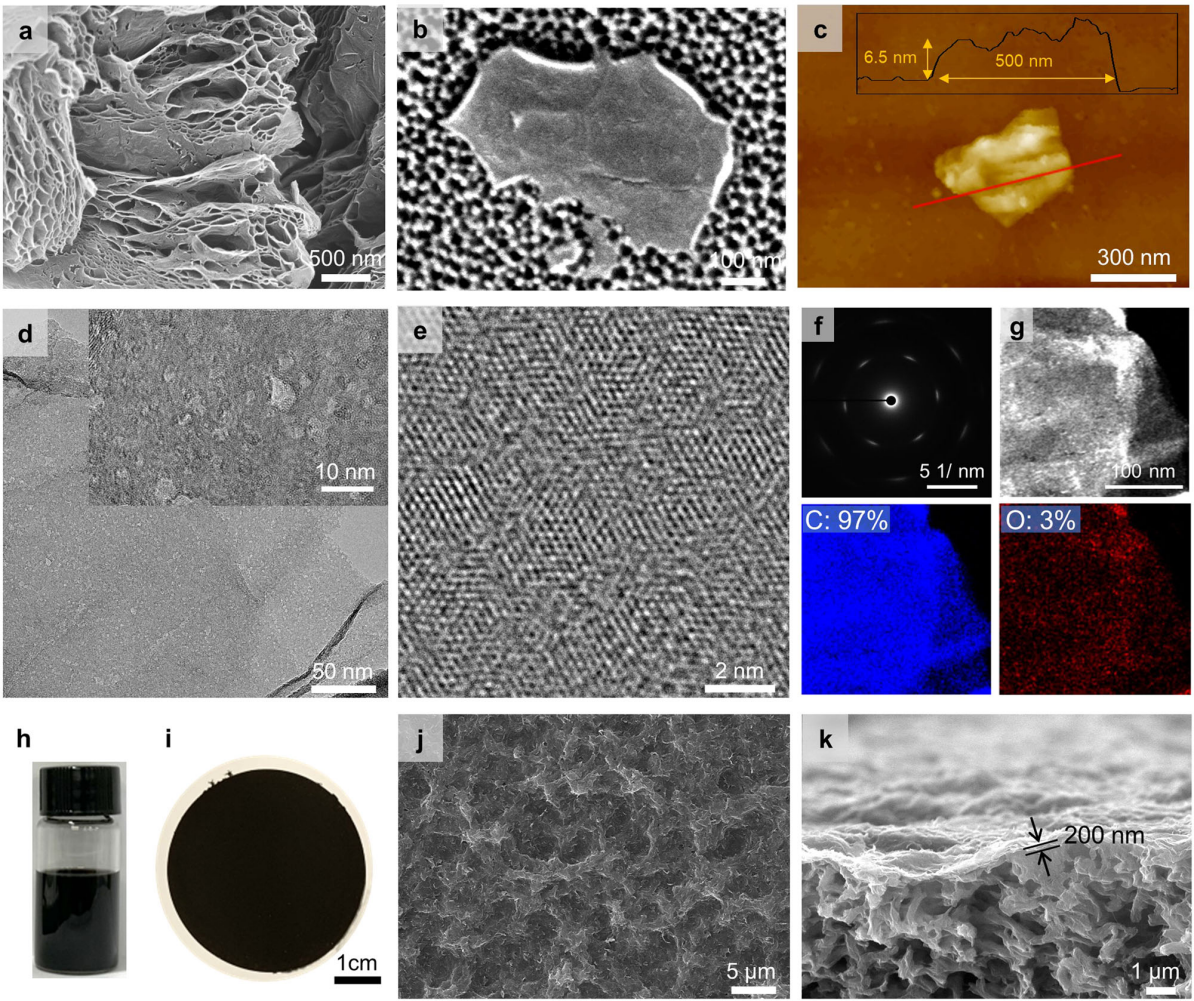
Structure of sp2-enriched nanoporous graphene and its membrane. **a**, **b** SEM images of MNG particle and exfoliated nanosheet. **c** AFM image of MNG nanosheet and its height profile. **d**, **e** HR-TEM images of MNG. **f** Corresponding selected area electron diffraction (SAED) pattern of MNG obtained from **d**. **g** TEM and corresponding EDS mapping images. The numbers represent the atomic weight percentages. **h**, **i** Photographic images of MNG dispersion in NMP at 0.5 mg/mL of concentration and MNG membrane. **j**, **k** Top-view and cross-sectional SEM images of MNG membrane on nylon support.

Figure 2a, b depicts the SEM images of MNG particles and exfoliated nanosheet, respectively. As-prepared GO sheets were densely stacked, whereas the NG layers were expanded during thermal activation by CO and CO_2_ gas emissions (Supplementary Fig. 1)^18^. The expanded graphene layers maintained a hierarchical structure after microwave treatment, but frequent porous defects were observed on the graphene surface, which can be attributed to the decomposition of oxygen-containing groups and sp^3^ carbons caused by microwave-induced local high temperatures (Fig. 2a). Figure 2b illustrates that the exfoliated MNG flake exhibits a lateral size of several hundred nanometers and a thickness of ~6 nm, revealed using atomic force microscopy (AFM) and corresponding height profile (Fig. 2c). Strong inter-sheet connections via van der Waals interaction or generation of intersheet chemical bonding appear to impede single-layer exfoliation of MNG sheets, which were mostly produced as multilayered sheets^29,30^. In optimizing microwave strength, more defects were observed to appear on the graphene plane with increasing microwave power (Supplementary Fig. 2) and 700 W was selected as the optimal condition to generate dense nanopores and restore sp^2^ domains. The treatment duration was maintained within a few seconds to preserve the graphene structure, which would otherwise burn.

As illustrated in Fig. 2d–f, high-resolution TEM images, and corresponding SAED and atomic mapping images were acquired to directly visualize the crystalline structure of the MNG flake. Although GO possessed numerous oxidized regions with partial sp^2^ regions, the oxidized regions were eliminated after thermal activation (Supplementary Fig. 3). However, because the activation temperature (200 °C) is too low to induce recrystallization of sp^3^ carbon, amorphous sp^3^ regions were observed and low crystallinity was confirmed by the broad hexagonal SAED pattern in Supplementary Fig. 3. On the other hand, the TEM images of MNG (Fig. 2d) show abundant nanopores and the high-resolution TEM image (Fig. 2e) reveals a hexagonal lattice structure of graphene, indicating the recovery of sp^2^ carbon bonds from amorphous sp^3^ carbons following microwave treatment. Sharp hexagonal patterns in the corresponding SAED pattern of MNG corroborate the microwave-induced transformation of sp^2^ domains (Fig. 2f). However, the SAED pattern is not as sharp as that of graphite or single-layer graphene grown by chemical vapor deposition owing to dense nanopore formation^31^. Energy dispersive spectroscopy (EDS) mapping images for carbon and oxygen (Fig. 2g) further demonstrate that oxygen groups were effectively eliminated by microwave treatment, as evidenced by the low oxygen quantities (3%).

Figure 2h depicts a photograph of the MNG solution dispersed in NMP at a concentration of 0.5 mg/mL using sonication and centrifugation. The MNG solution was filtered on commercial nylon supports (200 nm pore size) to fabricate membranes (Fig. 2i). Because of the high degree of reduction, the membrane color was as dark as graphite paper. Figure 2j, k displays the SEM images of uniform MNG layers with 200 nm thickness. Defective structures such as cracks and aggregated particles were undetected. In addition, the membrane was flexible and delamination of graphene layer was not observed as shown in the bending test (Supplementary Fig. 4), indicating good adhesion between graphene and polymer support.

### Effect of pore structure on the degree of microwave reduction

**Fig. 3:**
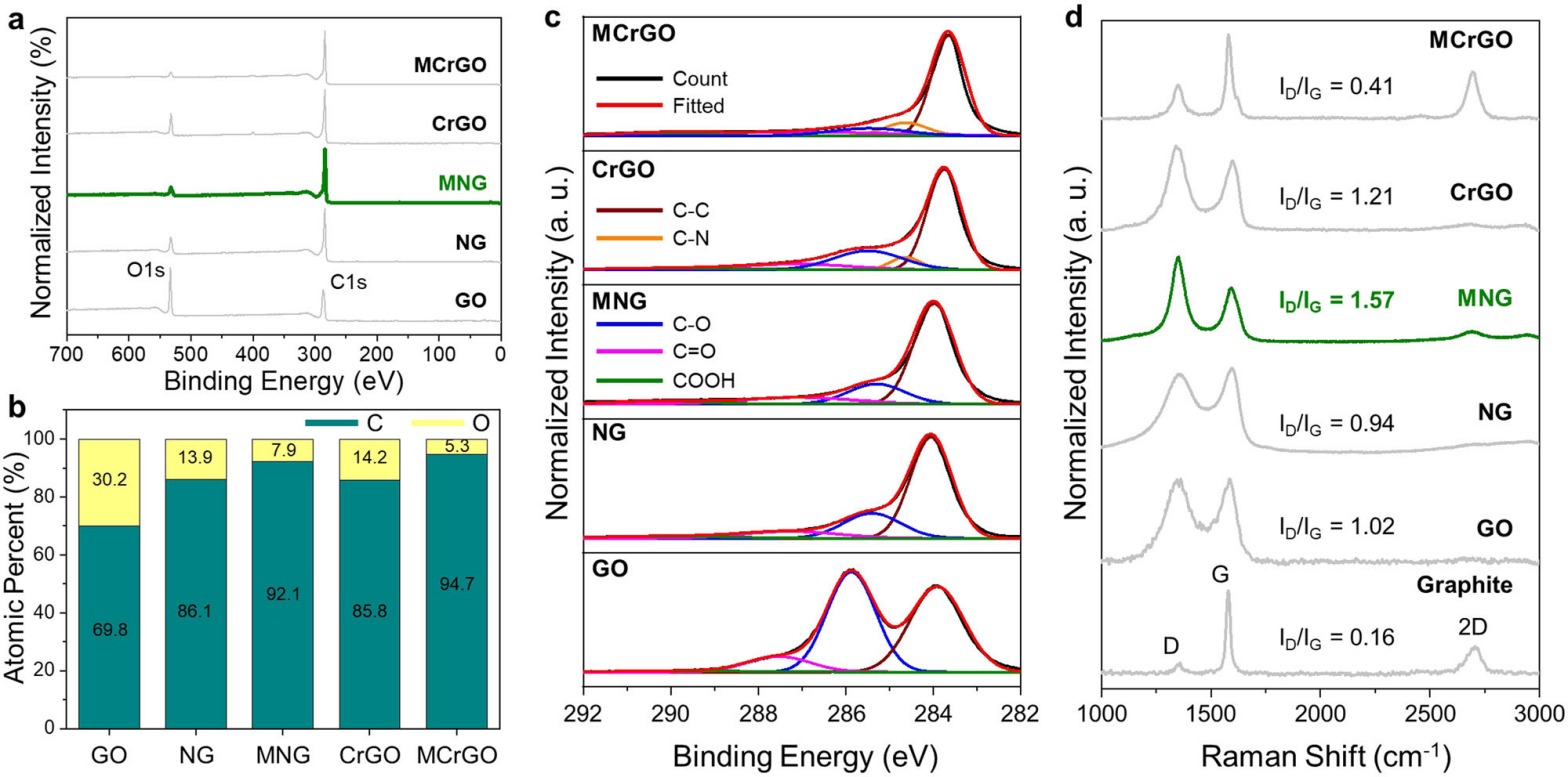
Chemical structure of graphene materials. **a** XPS survey scan, and **b** corresponding atomic percentage of GO, NG, MNG, CrGO, and MCrGO. **c** XPS C1s spectra and **d** Raman spectra of GO, NG, MNG, CrGO, and MCrGO. Raman spectra were measured using an excitation wavelength of 532 nm.

XPS and Raman spectroscopy investigation were conducted to analyze the effects of pore activation and microwave treatment on the crystalline structure of nanoporous graphene (Fig. 3). First, the chemical structures of graphene materials were investigated using XPS as depicted in Fig. 3a–c and Supplementary Fig. 5. The XPS survey scan and corresponding atomic percentage of samples (Fig. 3a, b) exhibit a reduced oxygen atomic percentage from 30.2% for GO to 7.9% for MNG due to the sequential thermal and microwave treatments. Chemically reduced graphene oxide (CrGO) with hydrazine exhibited a reduced oxygen atomic percentage of 14.2%, which further decreased to 5.3% after microwave treatment (MCrGO, microwave-treated chemically reduced graphene oxide). MNG has a slightly greater oxygen atomic percentage than MCrGO because NG comprised of a defective porous structure that is difficult to recrystallize. In addition, defective graphene is well known to exhibit low electrical conductivity, which may decrease the local temperature of graphene under microwave irradiation^28^.

The chemical bonds of graphene were further investigated as depicted in XPS C1s spectra in Fig. 3c and Supplementary Figure 5. GO exhibits oxygen-containing groups at C–O (285.9 eV), C = O (287.5 eV), and COOH (289.8 eV). Due to the decomposition of oxygen-containing groups, the C–O peak intensity significantly decreased after the first thermal annealing. The dominant C–C peak of MNG, generated from a defective amorphous carbon structure, was observed after microwave treatment. While the C1s spectrum shape of CrGO is similar to that of MNG, CrGO, and MCrGO primarily exhibited a C–C peak and an additional C–N (284.6 eV) peak originated from hydrazine. As shown in Fig. 3a, the atomic nuclei scans reveal low amounts of oxygen-containing groups in MCrGO, reconfirming a higher degree of reduction than MNG. The reduction of oxygen-containing groups was further confirmed by the FT-IR spectra (Supplementary Figs. 6–7), revealing the significantly decreased peak intensities related to oxygen-containing groups after thermal activation and microwave treatment. As depicted in Supplementary Fig. 6b, the peak intensities decreased with increasing microwave power owing to the enhanced decomposition of oxygen-containing groups at higher local temperatures. The low weight loss of MNG and MCrGO during the TGA test further validates the substantial reduction degree of graphene with microwave treatment (Supplementary Fig. 8), indicating the suppressed thermal decomposition of graphenes after microwave treatment. The contact angles of solvents (Supplementary Fig. 9) exhibited enhanced hydrophobic and oleophilic properties of the MNG membrane than graphene oxide owing to the low proportion of oxygen-containing groups.

Raman spectroscopy was employed to investigate the crystalline structure (Fig. 3d) because the presence of sharp *D*, *G*, and *2D* bands straightforwardly indicates the presence of long-range ordered sp^2^ carbon domains^32^. While directly visualized TEM images in Fig. 2 confirm the presence of ordered carbon crystals, the relatively large beam size (several micrometers in diameter) of Raman spectroscopy is more informative and reveals the crystal structure on a large scale. The *D*-band/*G*-band intensity ratio decreased from 1.02 for GO to 0.94 for NG owing to the decomposition of oxygen-containing groups following thermal activation. The broad *D*-band width of NG indicates amorphous sp^3^ carbon formation^33^. However, the Raman spectrum of MNG (shown in green) reveals a sharp *D*-band and the existence of a *2D* peak, indicating sp^2^ carbon recovery by microwave treatment^25^. Interestingly, the *D*/*G* intensity ratio of MNG significantly increased to 1.57 owing to dense nanopore formation and high concentration of carbon edges in nanopores^34,35^. The spectra shapes are identical to that of nanoporous graphene produced by etching CVD graphene with plasma or ion beam exposure^35^. The *D*-band intensity increased with increasing microwave power, possibly owing to the enhanced generation of defective pores (Supplementary Fig. 10). The *D*/*G* intensity ratio of MNG is remarkably higher and the *2D* peak intensity is lower than that of MCrGO, indicating that the NG pore structure hinders carbon crystallization compared to CrGO^36^. Because GO is unreactive towards microwave owing to its insulation properties, a comparison with neat GO was not performed. Moreover, the average size of sp^2^ domains was estimated using the Raman spectra of the samples (Supplementary Fig. 11)^37^. The sp^2^ crystallite size of MNG was calculated to be 13.5 nm, which is less than the size of GO (19.1 nm) and NG (20.7), implying that the sp^2^ domain size decreased throughout the thermal annealing and microwave treatment, despite the increasing density of sp^2^ domains.

Nanopore generation was also confirmed by N_2_ adsorption–desorption isotherms and corresponding pore size distribution (PSD) by Barrett–Joyner–Halenda (BJH) model (Supplementary Fig. 12 and Supplementary Table 1). Significant N_2_ adsorption by NG at relative pressures <0.1 indicates the presence of micropores^38^. MNG adsorbs more N_2_ than NG, and the PSD reveals MNG pore size to be <3 nm. Furthermore, the Brunauer–Emmett–Teller (BET) surface area increased from 306 m^2^/g for NG to 419 m^2^/g for MNG. These results indicate additional nanopore formation by microwave treatment. Based on the preceding observations, it is concluded that nanoporous graphene with high crystallinity can be successfully prepared using sequential thermal activation and microwave treatment, notwithstanding the presence of a small proportion of sp^3^ carbon or oxygen.

### Interlayer structure and swelling behavior in solvents

**Fig. 4:**
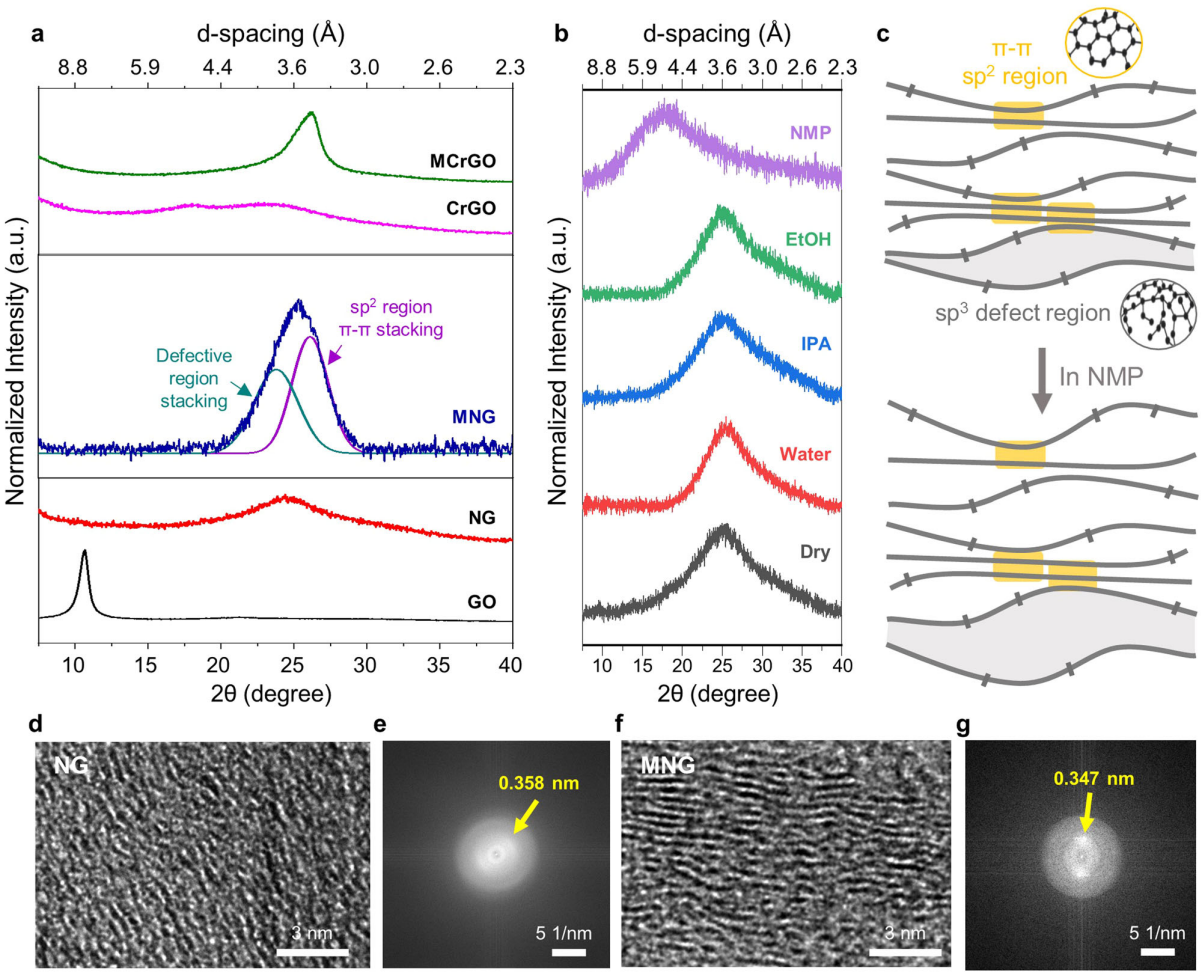
Interlayer structure of graphene materials. **a** Normalized XRD patterns of GO, NG, MNG, CrGO, and MCrGO. **b** XRD patterns of MNG membrane after immersion in various solvents. **c** Schematic structure of MNG membrane in dry-state and after swelling in a good solvent. **d**–**g** Cross-sectional HR-TEM images of NG (**d**, **e**) and MNG (**f**, **g**), respectively, and corresponding FFT images.

X-ray diffraction (XRD) patterns were obtained from graphene films to elucidate the interlayer structure of graphene after thermal activation and microwave treatment, as depicted in Fig. 4a. The d-spacing reduced from 8.3 Å for GO to 3.6 Å for NG owing to the removal of oxygen-containing groups after thermal activation^18^. The NG diffraction peak is broader than that of graphite owing to the less-ordered structure of NG resulting from the presence of amorphous sp^3^ carbons on the graphene plane^18^. After microwave treatment, the d-spacing of MNG (~3.5 Å) reduced below that of NG (3.6 Å) owing to enhanced reduction and increased crystallinity, and the d-spacing remained identical regardless of microwave power (Supplementary Fig. 13). Due to the presence of both sp^2^ regions and sp^3^ regions in the basal plane of MNG, a diffraction peak of MNG is considered to be composed of two patterns: defective region stacking and pi-pi stacking of sp^2^ regions^39^. This assumption is corroborated by the MCrGO peak, which exhibits a less broad and right-shifted pattern because MCrGO is primarily structured with sp^2^ carbons resulting from a higher degree of reduction and the absence of dense nanopores.

The XRD patterns in solvents were further analyzed to determine the interlayer spacing variations in the MNG membrane in response to solvent type (Fig. 4b). The d-spacing values of the MNG membrane are similar in water, ethanol, and IPA, which are known to be poor solvents for graphene dispersion, and these values are near to that of the MNG membrane in the dry state. In contrast, the d-spacing value was significantly increased in NMP which is a well-known good solvent for graphene dispersion^40^. The interlayer spacing variation in solvents is illustrated in Fig. 4c. The MNG membrane exhibits a combined structure with defective sp^3^-stacked regions and graphitic sp^2^-stacked regions. Particularly, the spacing composed of defective sp^3^ regions is more easily intercalated by solvent molecules than the narrow spacing of sp^2^ regions. Therefore, the interlayers may further swell in good solvents. In contrast, the interlayer structure with sp^2^-stacked regions could be rigidly maintained owing to the pi–pi interactions of sp^2^ regions, which is crucial for fabricating stable membranes in solvents.

As depicted in Fig. 4d–g, cross-sectional HR-TEM images of the MNG membrane were obtained to reveal its interlayer structure. Both samples were prepared using the focused ion beam technique. The TEM images of both NG and MNG membranes depict the stacked graphene layers; however, the MNG membrane exhibits better aligned and compactly stacked graphene layers. The enhanced ordered structure of the MNG membrane was further confirmed by the FFT patterns denoted by yellow arrows, indicating a broader arc pattern from the NG sample with larger d-spacing.

### OSN performance of MNG membrane in various solvents

**Fig. 5:**
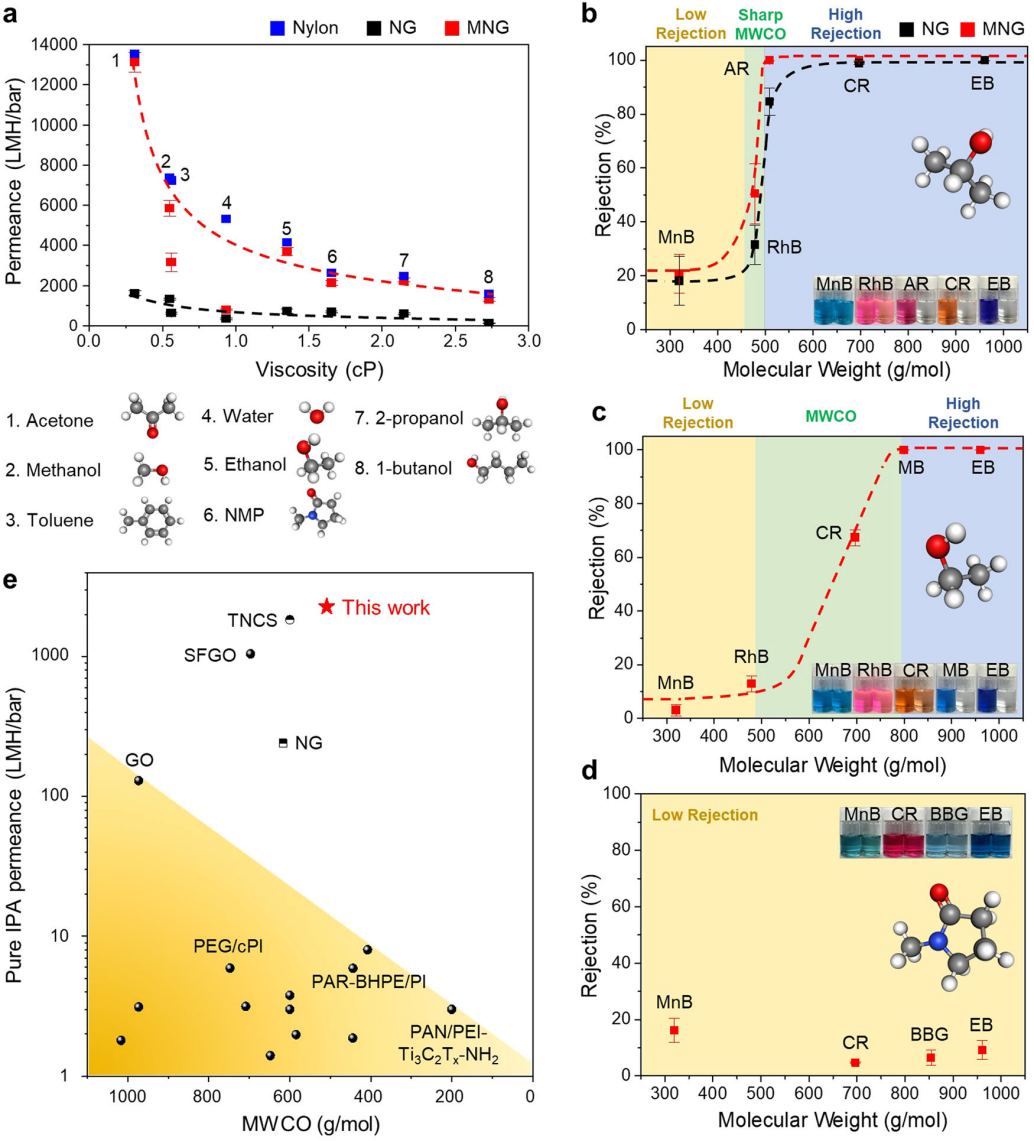
Organic solvent nanofiltration performance of MNG membrane. **a** Permeance of pure organic solvents across 200 nm thick NG and MNG membranes as a function of viscosity. **b–d** Nanofiltration performance of MNG membranes tested with several organic solvents and dye molecules including methylene blue (MnB), rhodamine B (RhB), acid red 1 (AR), congo red (CR), methyl blue (MB), brilliant blue G (BBG), and evans blue (EB) at 3 bar: IPA (**b**), ethanol (**c**), NMP (**d**). **e** Comparison of IPA nanofiltration performances. All data points were averaged from at least three samples.

The membrane was tested as depicted in Fig. 5 to estimate the nanofiltration performance of the MNG membrane in various organic solvents. The permeance of various pure organic solvents was initially evaluated (Fig. 5a and Supplementary Table 2) at 3 bar using the 200 nm thick NG and MNG membranes (Fig. 2k and Supplementary Fig. 14). The MNG membrane demonstrated ultrafast permeances for various organic solvents, ranging from 13117 LMH/bar for acetone to 1300 LMH/bar for 1-butanol, which are surprisingly near to the solvent permeances of a bare nylon support. In summary, the solvent permeance was inversely proportional to the solvent viscosity. The solvent permeance in 2D lamellar membranes can be calculated theoretically by using classical Hagen-Posieuile (HP) equation^41^. The permeance of MNG membrane (2278 LMH/bar of IPA) is exceptionally higher than the theoretical values as shown in Supplementary Fig. 15. A possible reason can be the smoother surface of highly reduced nanoporous graphene with dense sp^2^ carbon regions, making the transport of ordered solvent molecules easier in the nanochannels. In addition, low friction at graphene surface by low oxygen groups and sp^2^ regions can be favorable for the solvent transport, as observed in the fast water transport through supported graphene nanochannel^41,42^.

Remarkably, toluene and water exhibited relatively low permeance. The low permeance of toluene is expected to arise from the large molecular size of toluene, resulting in hindered transport within the interlayers^43^. Moreover, a lower water flux than other solvents could result from the hydrophobic surface of MNG, which is unfavorable to water sorption. Several previous studies had either expected the selective permeation of alcohol or nonpolar solvent through hydrophobic nanoporous graphene channels^44^, or the promotion of hydrogen bonding with water by residual oxygen-containing groups, thereby hindering water molecule diffusion^45,46^. In addition, it is emphasized that the MNG membrane exhibited significantly higher permeances than the NG membrane owing to the increased production of nanopores and the smoother surface comprised of dense sp^2^ carbon regions. Detailed mechanisms of enhanced organic solvent transport in graphene nanochannel are subsequently discussed herein using computational simulations.

Figure 5b–d illustrates the OSN performance of the MNG membrane in IPA, ethanol, and NMP with probe dye molecules ranging in size from 300 to 1000 Da. The tests were conducted at 3 bar and 10 mg/L of dye concentration (Supplementary Fig. 16). As depicted in Fig. 5b, the MNG membrane exhibited nearly 100% high rejection rates for EB (961 Da), CR (697 Da), and AR (509 Da), whereas smaller molecules exhibited lower rejection rates such as RhB (479 Da, 50.5%) and MnB (320 Da, 20.7%). The NG membrane demonstrated lower rejection rates than the MNG membrane below 600 Da, such as AR (84.6%), RhB (31.5%), and MnB (18.1%), indicating the enhanced rejection performance by the better ordered and narrower interlayers of MNG. The MNG membrane was evaluated in ethanol and NMP under identical conditions (Fig. 5c, d). Interestingly, the MWCO of the MNG membrane increased from 509 Da to 800 Da in ethanol; furthermore, the MNG membrane exhibited low rejection rates below 20% for all dyes in NMP. Because the membrane exhibited stability in NMP, membrane damage cannot account for the low rejection rate (Supplementary Fig. 17). According to the XRD patterns of swollen MNG membranes, the low rejection rates in ethanol and NMP may be attributable to the enlarged interlayer spacing in ethanol and NMP (Fig. 4b). Moreover, the smaller effective/solvated diameters of dyes in ethanol can cause low rejection rates^47^. These results indicate that the separation performance of MNG membrane can be altered in response to different solvent types. In addition to the aforementioned solvents, we also tested MNG membranes with acetone and methanol, which are also one of the most widely used solvents in organic chemistry (Supplementary Fig. 18). MWCO was around 450 Da in acetone and around 750 Da in methanol. We also confirmed that the retentate was concentrated after filtration due to the filtered dyes on feed side, indicating that the rejection occurred by molecular sieving rather than adsorption (Supplementary Fig. 19).

The OSN performance of the MNG membrane was compared to that of prior OSN membranes using the upper bound based on pure IPA permeance and MWCO (Fig. 5e and Supplementary Table 3). The MNG membrane exhibited an ultrafast pure IPA permeance of 2278 LMH/bar and the MWCO of 509 Da, which significantly exceeded the upper bound of previously reported OSN membranes. In addition, the MNG membrane performance in ethanol was compared to that of other OSN membranes (Supplementary Fig. 20 and Supplementary Table 4) and the MNG membrane stability was verified using an immersion test and long-term filtration test under cross-flow conditions (Supplementary Figs. 21–25). For 30 days, the MNG membrane proved extremely stable in various solvents including water, IPA, acid, and alkaline solutions (Supplementary Fig. 21). Furthermore, the membrane exhibited extremely stable rejection rates (>99%) for two days during the cross-flow filtration test (Supplementary Figs. 22–25), which is achieved by strong pi–pi interaction between MNG flakes. Moreover, any crack on the membrane surface was not observed after the filtration test even though a dye cake was formed due to the fast solvent permeance (Supplementary Fig. 24). In addition, the membrane showed excellent diafiltration performance with binary mixed dye mixture feed under cross-flow conditions, maintaining high rejection rates of EB above >99%, while Methyl orange (MO) rejection rate was ~0% (Supplementary Fig. 25).

### Multiple mixture separation by MNG membrane

**Fig. 6:**
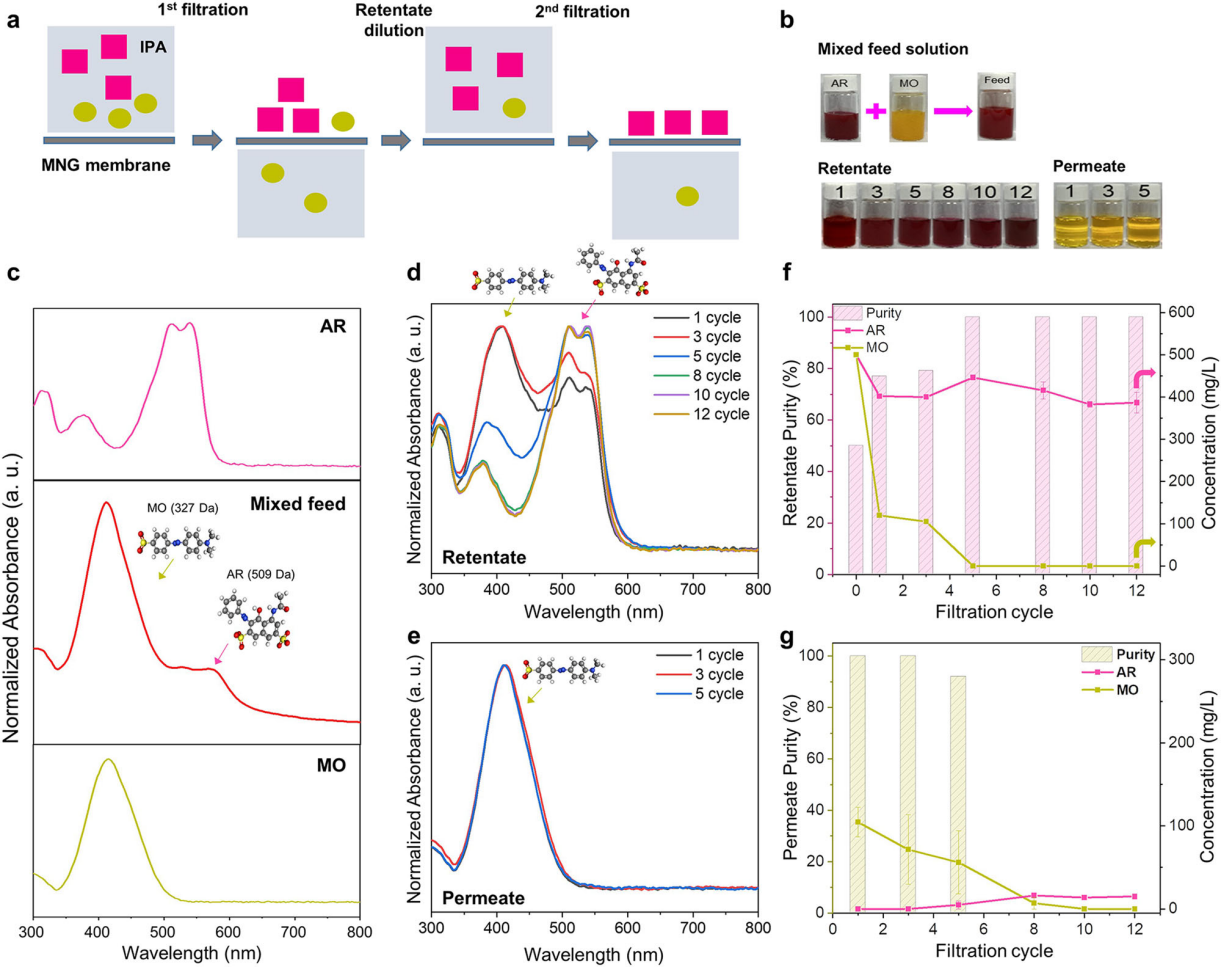
MNG membrane for binary dye mixture separation under dead-end filtration. The test was conducted at 3 bar and dilution was conducted with 100 mL of IPA. The total concentration was 1000 ppm in IPA and the weight ratio of the dyes was 1:1. **a** Schematic illustration of binary dye mixture separation. The colored square and circle are dye molecules. **b** Photographs of feed, re-dispersed retentate, and permeate solutions after each filtration cycle. **c** UV–Visible absorbance spectra of AR, MO, and their mixture solutions in IPA. **d**, **e** UV–Visible absorbance spectra of re-dispersed retentate and permeate solutions after each filtration cycle. **f**, **g** Purity and concentration of AR and MO in the re-dispersed retentate and permeate solutions after each filtration cycle.

To explore the feasibility of separation for mixed molecules, binary mixture separation tests were conducted using the MNG membrane under a dead-end condition (Fig. 6 and Supplementary Fig. 26). The test was conducted at 3 bar with a total concentration of 1000 ppm in IPA for the binary feed solution. The feed solution was filtered through the MNG membrane, following which the retentate was re-dispersed and re-filtrated. The process was repeated several times to perfectly separate the binary mixture (Fig. 6a). After initial filtering, the yellow solution (MO, 327 Da) was permeated without the dark red solution (AR, 509 Da), and the re-dispersed retentate solution darkened as the yellow solution of MO was completely extracted (Fig. 6b). In the UV–Vis spectra (Fig. 6c–e and Supplementary Fig. 27), while the mixed feed solution contains two absorbance peaks of AR and MO (Fig. 6c), the permeate solutions exhibited a single peak of smaller MO by the fifth cycle (Fig. 6e). Furthermore, the re-dispersed retentate solution only exhibited a peak corresponding to larger AR after the fifth cycle, indicating the successful separation of most smaller MO molecules and the concentration of larger AR molecules based on size exclusion using the MNG membrane (Fig. 6d). Figure 6f, g exhibits the purity and concentration of AR and MO in feed and permeate side after each filtration cycle, indicating the separation of highly concentrated AR and MO with a high purity of nearly 100% on each side after five cycles of filtration. Most importantly, due to the ultrafast solvent permeance of the MNG membrane, a single filtration of 100 mL of solution was completed within a minute, indicating that the operation efficiency can be significantly greater than conventional OSN membranes or distillation.

**Fig. 7:**
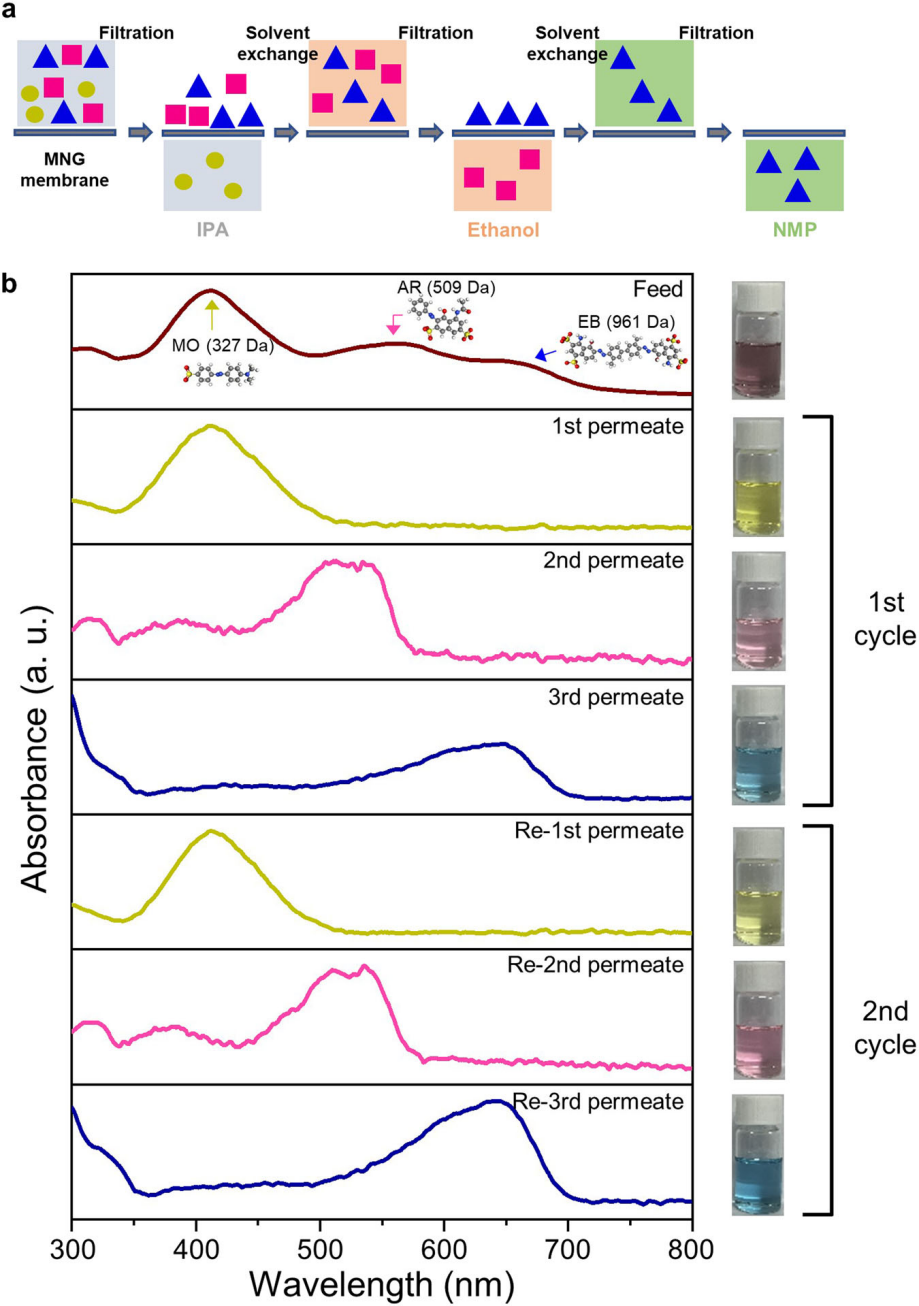
Switching MNG membrane performance for ternary dye mixture separation under dead-end filtration. **a** Schematic image of ternary dye mixture separation. The colored square, circle, and triangle are dye molecules. **b** UV–Visible absorbance spectra and photographic images of feed and permeate solutions. The total concentration was 10 ppm in IPA and the weight ratio of the dyes was 1:1:1. The test was conducted at 3 bar. The feed solvent was IPA. Ethanol and NMP were used for second and third filtration respectively.

A membrane with switchable molecular separation properties is verily required to purify multiple mixtures with single equipment because separation processes can be accomplished without membrane replacement. To demonstrate the switchable molecular separation property of the MNG membrane, the filtration test was conducted at 3 bar with a feed of ternary dye mixtures containing MO (327 Da), AR (509 Da), and EB (961 Da) in IPA (Fig. 7) and the separation was conducted by solvent exchange to exploit the solvent-dependent switchable interlayer structure of MNG (Fig. 7a). The brown feed solution exhibits three absorbance peaks of MO, AR, and EB as shown in Fig. 7b. After the initial filtration, the yellow solution (MO) was permeated with only the MO absorbance peak as MO is below the 509 Da of MWCO in IPA. During the second filtration, the pink solution (AR) was permeated with an AR peak as AR is below the 800 Da of MWCO in ethanol. Finally, the blue solution (EB) was completely permeated in NMP with an EB peak, demonstrating that the ternary molecular mixtures were successfully separated by solvent exchange with the MNG membrane. Furthermore, the filtration process was repeated with the ternary mixture feed solution to confirm the reversible switching properties. The MNG membrane exhibited the same result after the second test as depicted in the UV–Visible absorbance spectra and photo images of permeate solutions. The cycle test confirmed that the interlayer structure of the MNG membrane is switchable based on the solvents and that the separation performance is reversible. For polymeric membranes, the changes of MWCO by swelling of polymer in organic solvents have been widely reported. However, the MWCO changes of polymeric membranes are normally not fast and not precise as we are reporting with our graphene membrane. We want to emphasize that the switchable separation property of MNG is attributable to the fast interlayer structure modulation of graphene by solvents. Indeed, the solvent exchange during the test was conducted immediately after filtering solvent, so this phenomenon is more close to switching of MWCO by solvent type while solvent swelling in the graphene channel is the origin of the switching phenomena.

**Fig. 8:**
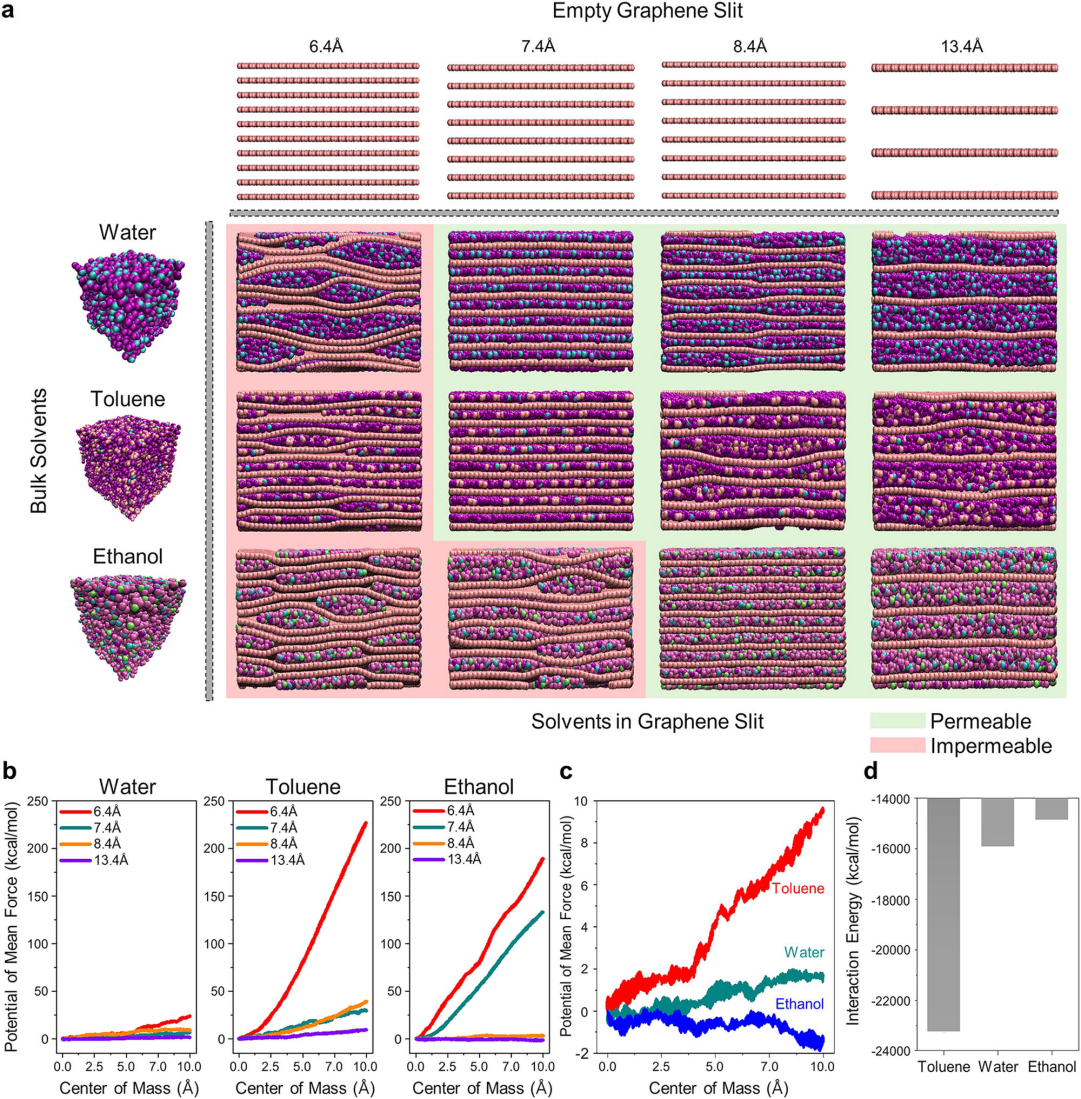
Steered MD simulation of solvent permeation in graphene layers. **a** Snapshots of solvents (water, toluene, and ethanol) passing through graphene layers with various d-spacings. **b** Potentials of mean force displayed as a function of the center of mass of solvent molecules for the steered MD simulations described in **a**. **c** Potentials of mean force of water, toluene, and ethanol at a d-spacing of 13.4 Å. **d** Interaction energy between graphene layers and solvents within the d-spacing of 13.4 Å.

A steered MD simulation approach was employed to investigate the change in the thermodynamic free energy, i.e., the potential of mean force, as a solvent permeates through the graphene layers. This thermodynamic quantity represents the solvent molecule permeability within the graphene layers. Figure 8a depicts snapshots pertaining to the observed behaviors of solvent molecules and graphene layers during the steered MD simulations. The model utilized herein is based on a pristine graphene layer. While graphene oxide or reduced graphene oxide possess oxygen-containing groups or defective sp^3^ carbon regions, which are known to affect solvent permeation through interaction or steric hindrance^23,24^, it is straightforward to understand intrinsic solvent permeation through the graphene channel when pristine graphene spacing is considered. In addition, the effect of nanopores is disregarded because nanopores with dimensions of several nanometers are sufficiently large to permit solvent permeation, and the primary resistive channel is the narrow interlayer spacing of stacked graphene.

As depicted in the snapshots, the solvent molecule permeability strongly depends on the solvent identity and interlayer spacing. Water and toluene tend to cluster at a d-spacing of 6.4 Å, exhibiting multiple distributions of locally overlapping graphene layers. This can be attributed to the Connolly-analyzed diameters of water (3.37 Å) and toluene (3.83 Å) molecules, which are larger than the accessible interlayer spacing (3.0 Å) calculated under the condition of the van der Waals diameter (3.4 Å) of graphene carbon (Supplementary Fig. 28). Therefore, the graphene layers swell to create a local space for the solvent molecules, while the graphene layers locally overlap to compensate for the swollen space. The Connolly-analyzed diameter of toluene is assumed to be identical to methane because aromatic rings of toluene molecules are positioned parallel to the graphene layers. Using identical reasoning, the solvent molecules become well distributed throughout entire graphene layers as the layer spacing increases at d-spacings of 7.4, 8.4, and 13.4 Å, which correspond to the accessible interlayer spacings of 4.0, 5.0, and 10.0 Å, respectively (Supplementary Fig. 28). No local overlapping of the graphene layers is observed at these interlayer spacings. Consequently, these solvents have significantly higher potentials of mean force at the layer spacing of 6.4 Å compared to larger interlayer spacings, as shown in Fig. 8b, indicating the highest resistance against the solvent permeation at the layer spacing of 6.4 Å. Using the same reasoning, the minimum layer spacing at which ethanol molecules homogeneously pass through non-overlapping graphene layers is determined to be 8.4 Å, considering the Connolly-analyzed diameter (4.79 Å) of ethanol. Therefore, the potentials of mean force at the interlayer spacings of 6.4 and 7.4 Å are significantly greater compared with those at layer spacings of 8.4 and 13.4 Å. Conclusively, these results combinedly imply that the permeability of solvent molecules would be significantly enhanced at interlayer spacings with a free volume that can accommodate solvent molecules without any swelling behaviors of graphene layers, as indicated in green in Fig. 8a.

Further comparison of potentials of mean force at the interlayer spacing of 13.4 Å for all three types of solvents reveals that toluene has the highest resistance to solvent permeation, while ethanol exhibits the least resistance (Fig. 8c). As depicted in Fig. 8d, the simulated interaction energies between graphene layers and solvent molecules demonstrate that the aforementioned difference in the potentials of mean force would result from the difference in the interaction energy, which follows the order of toluene < water < ethanol. Note that a larger negative interaction energy value results in stronger interaction between graphene layers and solvent molecules. Consequently, the van der Waals-based strongest interaction between graphenes and aromatic rings in toluene molecules leads to the strongest resistance against the solvent permeation, whereas stronger hydrogen bonding characteristics in water molecules would result in a stronger resistance of water relative to ethanol against solvent permeation. The simulation results imply that a larger interlayer spacing than the size of the solvent is critical to achieving high solvent permeation through graphene nanochannels, and that permeation can be further enhanced by modifying the graphene surface with low interaction energy while maintaining the same interlayer spacing. It is expected that the turbostratic nanochannel of the proposed graphene membrane is one of the reasons for an ultrafast solvent flux, despite the fact that the swelling degree of different solvents is generally uncontrollable in experiments, unlike the simulation model.

## Discussion

This study demonstrates the easy fabrication of nanoporous graphene with high crystallinity by combining thermal activation and microwave treatment. Previous reduction methods, such as chemical reduction, are unable to restore the hexagonal lattice of defective and oxidized graphene. However, microwave treatment is highly effective for regenerating hexagonal carbon lattice as maintaining nanoporous structure. In addition, the treatment process only requires a few seconds, which is desirable for the future scalability of nanoporous graphene membrane. Using graphene as a building block, a nanoporous multilayer graphene membrane with ultrafast solvent permeation and switchable rejection performance was developed. By recovering the sp^2^ carbon structure from sp^3^ carbons through microwave treatment, an enhanced ordered interlayer structure with dense nanopores can be produced. Consequently, the membrane exhibited flexible and narrow MWCO owing to the swelling degree of interlayers depending on the solvent type. Furthermore, the membrane was demonstrated to feasibly perform the separation of multiple molecular mixtures, which can be utilized for chemical extraction and concentrations in various application fields, including petrochemicals and pharmaceuticals. Further, the proposed graphene preparation methods can be applied to sensors, energy storage devices, adsorbents, and electrocatalysis, all of which require a high surface area and electrical conductivity.

## Methods

### Synthesis of NG powder

GO powder was prepared using a modified Hummers’ method as described in our previous studies^48^. First, 2 g of graphite powder was dissolved in 150 mL of sulfuric acid. Second, 7 g of potassium permanganate (KMnO_4_) was slowly added to the graphite dispersion and stirred at 35 °C for 5 h in a water bath. After stirring, 200 mL of deionized (DI) water was slowly added to the dispersion in an ice bath. Subsequently, 100 mL of hydrogen peroxide (H_2_O_2_) was poured into the solution. The products (GO) were washed and filtered several times with DI water to remove the remaining chemicals. Finally, GO powder was obtained by freeze-drying. The prepared GO powder was loaded into a quartz tube, which was then placed into a furnace set to 200 °C. The GO powder was thermally annealed in the furnace at 200 °C under ambient conditions for 5 min to activate nanopores. The activated powder, NG, was collected after cooling to room temperature in air.

### Production of MNG membrane

The as-prepared NG powder was placed into a commercial microwave oven. The NG powder was treated at 700 W for 2 s. The resultant MNG powder was collected and dissolved at a concentration of 0.5 mg/mL in NMP. The solution was dispersed using a horn-sonicator (Sonic VCS 505) in an ice bath for 300 min. The obtained MNG solution was centrifuged at 5400 × *g* (7000 rpm) for 30 min to remove large and undispersed particles, and the dispersed MNG solution was collected from the supernatant. The as-prepared MNG solution was diluted with deionized water and subsequently filtered through nylon support (Whatman, 0.2 µm pore size). After filtration, the MNG membranes were dried in an oven at 60 °C overnight to eliminate any leftover solvents.

### Binary dye mixture separation using a dead-end system

100 mL of feed solution containing methyl orange (MO; 327.33 g·mol^−1^) and acid red 1 (AR; 509.43 g·mol^−1^) dyes (1:1 weight ratio) at a total concentration of 1000 mg/L in IPA was filtered through an MNG membrane using a homemade dead-end cell until the entire filtration of the solvent. Next, 100 mL of pure IPA was introduced to the dead-end cell to re-disperse the retentate, and the solution was filtered again. The process was repeated 12 times. The concentrations of feed, permeate, and re-dispersed retentate solutions were calculated using UV–Vis spectroscopy.

### Ternary dye mixture separation using a dead-end system

Under the dead-end condition, 100 mL of feed solution containing methyl orange (MO; 327.33 g·mol^−1^), acid red 1 (AR; 509.43 g·mol^−1^), and Evans blue (EB; 960.8 g·mol^−1^) dyes (1:1:1 weight ratio) at the total concentration of 10 mg/L in IPA was filtered through the MNG membrane. After the completion of solvent filtration, 100 mL of pure ethanol was added to the dead-end cell to re-disperse the retentate. Subsequently, the solvent was filtered. After adding 100 mL of pure NMP to the cell to re-disperse the retentate, the solvent was filtered again. This process of solvent exchange was repeated once more. The solvent exchange process was conducted immediately after each completion of solvent filtration.

### Computational methods

The molecular dynamics (MD) simulation approach was employed to investigate the permeation behaviors of solvent molecules passing through the graphene layers. First, each periodic simulation model was initially prepared to contain graphene layers with solvent molecules occupying space between the layers. Either toluene, water, or ethanol was introduced as a solvent in each simulation model. The prepared models were then equilibrated by a series of NVT (298 K) and NPT (298 K and 1 atm) ensembles for 5 ns to have graphene layers with a targeted layer spacing in which space between graphene layers was filled with solvent molecules. Four distinctive layer spacings, namely 6.4, 7.4, 8.4, and 13.4 Å, were introduced for each solvent case to identify suitable layer spacings for the facilitated solvent permeation. Nose–Hoover thermostat and barostat were used to control the temperature and pressure under the condition of the NPT ensemble, respectively. All these simulations were performed using the LAMMPS simulation package with the DREIDING force field^49,50^. Next, a steered MD simulation approach implemented in the LAMMPS simulation package was performed to predict free energy changes associated with the behaviors of solvent molecules passing through the graphene layers. Note that the steered MD simulation approach is suitable to understand the thermodynamic preference of the designated pathways of solvent molecules. During the steered MD simulations, a spring constant of 1000 kcal/mol/Å and pulling velocity of 10^−5 ^Å/fs were applied to solvent molecules in the simulation models while graphene layers were not presumed to be pulled out. The free energy profiles of the solvent molecules passing through the space between graphene layers during the steered MD simulations were recorded in form of the potential of mean force (PMF). All the steered MD simulations were performed under NPT (298 K and 1 atm) ensemble for 1 ns.

## Supplementary information

Supplementary Information

## Data Availability

The data supporting the findings of this study are available within the paper and Supplementary Information. Any additional detail can be requested from the corresponding authors.
