# Supplementary material for: Microwave-assisted design of nanoporous graphene membrane for ultrafast and switchable organic solvent nanofiltration
Source: Nat Commun. 2023 Feb 17;14:901. doi: 10.1038/s41467-023-36524-x (PMC9935848; doi:10.1038/s41467-023-36524-x)
Supplement: Supplementary file 1 — Supplementary Information [file 41467_2023_36524_MOESM1_ESM.pdf]

Supplementary Information

Microwave-Assisted Design of Nanoporous Graphene Membrane for Ultrafast and Switchable Organic Solvent Nanofiltration

Junhyeok Kang[1], Yeongnam Ko[2], Jeong Pil Kim[1], Ju Yeon Kim[1], Jiwon Kim[1], Ohchan Kwon[1], Ki Chul Kim[2], Dae Woo Kim[1,]*

[1]Department of Chemical and Biomolecular Engineering, Yonsei University, Yonsei-ro 50, Seodaemun-gu, Seoul (03722), Republic of Korea

[2]Department of Chemical Engineering, Konkuk University, Seoul, 05029, Republic of Korea

Supplementary Methods:

Materials. Graphite powder was purchased from Sigma-Aldrich. Potassium permanganate ($KMnO_4$) was purchased from Duksan. Sulfuric acid (H_2SO_4), Hydrogen peroxide (H_2O_2), and Hydrochloric acid (HCl) were purchased from Daejung. Acetone, methanol, toluene, ethanol, 1-methyl-2-pyrrolidone (NMP), isopropyl alcohol (IPA), and 1-butanol were purchased from Daejung. Evans blue (EB), brilliant blue G (BBG), methyl blue (MB), congo red (CR), acid red 1 (AR), rhodamine B (RhB), methylene blue (MnB) and methyl orange (MO) were purchased from Sigma-Aldrich. Hydrazine monohydrate and Sodium hydroxide (NaOH) was purchased from Sigma-Aldrich.

Preparation of CrGO and MCrGO powder. The prepared GO powder was dispersed in water at a concentration of 3 mg/mL. Hydrazine monohydrate (2.8 μL for 3 mg of GO, 35%, Sigma-Aldrich) was added to the GO solution. The solution was stirred at 200 rpm at 60 °C for 2 h in an ice bath. The resulting CrGO powder was filtered by washing and dried in an oven at 50 °C. For MCrGO powder, the CrGO powder was treated at 700 W for 2 s using a microwave oven, which is identical to the method employed for MNG powder.

OSN performance test using dead-end filtration. Various dyes including methylene blue (MnB; 319.85 g·mol^{-1}), rhodamine B (RhB; 479.0 g·mol^{-1}), acid red 1 (AR; 509.43 g·mol^{-1}), congo red (CR; 696.7 g·mol^{-1}), methyl blue (MB; 799.81 g·mol^{-1}), brilliant blue G (BBG; 854.02 g·mol^{-1}), and evans blue (EB; 960.8 g·mol^{-1}) were utilized as probe molecules to evaluate OSN performance. The dye solution concentration in IPA, ethanol, NMP, methanol, and acetone was 10 mg/L. The effective area of the membrane was 10.17 cm^2. The filtration tests were conducted at 3 bar which was controlled using the pressure of a nitrogen cylinder. Rejection and permeance were analyzed by collecting 15 mL of permeate to avoid the concentration polarization effect. The permeance (J; L m^{-2}h^{-1}bar^{-1}) and rejection rate (R; %)

were calculated using the following equations.

$$J = \frac{V}{tA\Delta P} \quad (1)$$

$$R = \frac{C_f - C_p}{C_f} \times 100 \quad (2)$$

Here, V (L) is the permeate volume, t (h) is the filtration time, A (m^2) is the effective area of the membrane, and ΔP (bar) is the applied pressure. C_f and C_p are the dye concentrations in the feed and permeate, respectively. The solution concentration was calculated using UV–Vis spectroscopy.

Long-term stability test using a cross-flow system. The effective area of the membrane was 7.1 cm^2. The feed solution including EB dye in IPA was a concentration of 10 mg/L with 1.7 L/min of flow rate. For the binary dye mixture, the total concentration of feed solution was 20 mg/mL. The cross-flow filtration test was conducted at 2 bar for 2 d. The retentate was circulated to a feed tank by a hydraulic pump. The rejection rate was measured using UV–Vis spectroscopy.

Characterization. SEM images were obtained using IT-500HR (JEOL Ltd., Japan). AFM analysis was conducted with NX-10 (Park Systems, South Korea). HR-TEM investigation was conducted using JEM-ARM 200F NEOARM (JEOL Ltd., Japan) at an accelerating voltage of 80 kV. The XPS data were acquired using K-alpha (Thermo U. K., USA). Raman spectra were obtained by Labram Aramis (Horriba Jovin Yvon, Japan) with an excitation wavelength of 532 nm. The XRD analysis was conducted using Ultima IV (Rigaku, Japan) with a beam source of Cu-Kα (λ = 1.5406 Å). N_2 adsorption isotherms were measured using BELSORP-mini II (BEL Japan) at 77K and the Barrett–Joyner–Halenda (BJH) method was used to obtain pore-size distribution. FT-IR spectra were measured using the Excalibur series FT-IR instrument (Diglab

co., Hannover, Germany). UV–Visible spectra were obtained using Ubi-490 (MicroDigital Co., Ltd., South Korea). TGA analysis was conducted using TGA55 (TA Instruments, USA) under an N_2 atmosphere at the scan rate of 20 °C/min. Contact angle measurement was conducted with Attension Theta Lite (Biolin Scientific, Sweden).

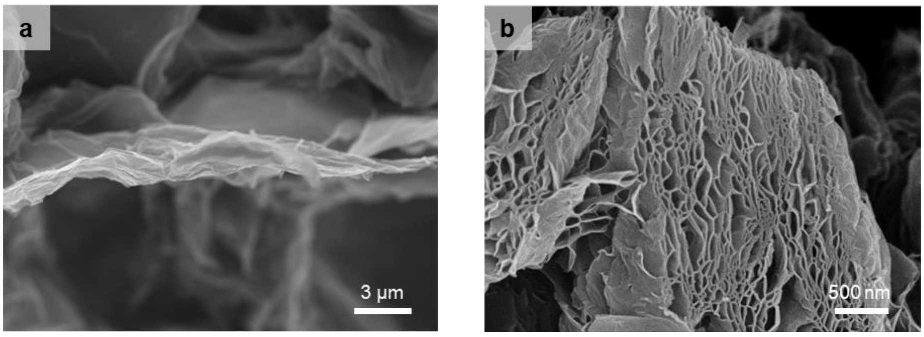

Supplementary Figure 1. a and **b**, SEM images of graphene oxide (GO) and nanoporous graphene (NG) powder prepared by thermal exfoliation.

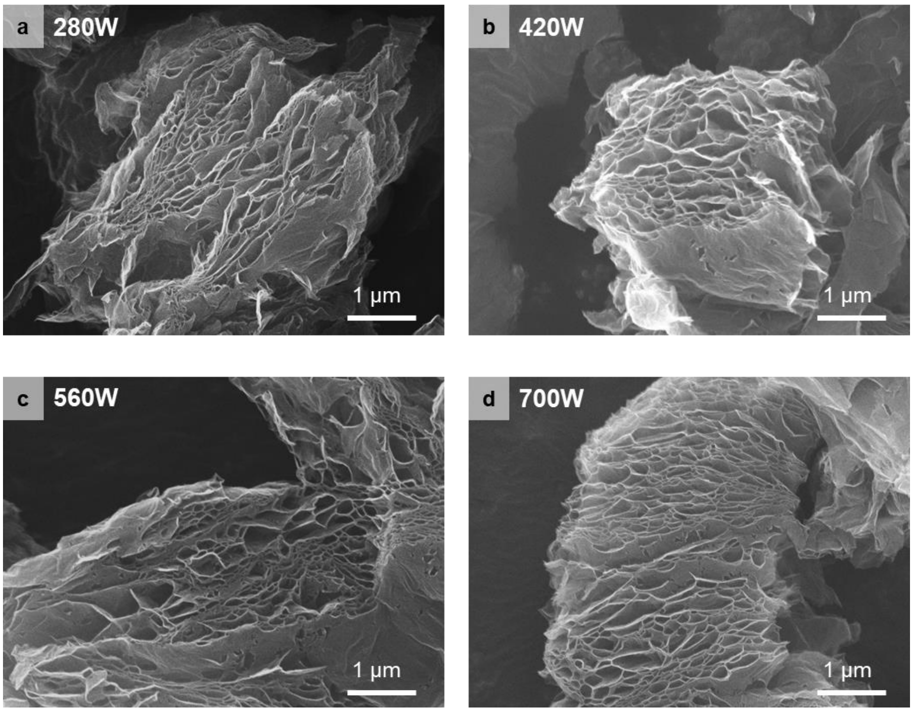

Supplementary Figure 2. a–d, SEM images of MNG at various microwave powers. All powders were treated for 2 s. The expanded layers were maintained after microwave treatment regardless of the microwave power. In addition, denser defects appeared on graphene surface as the microwave power increased.

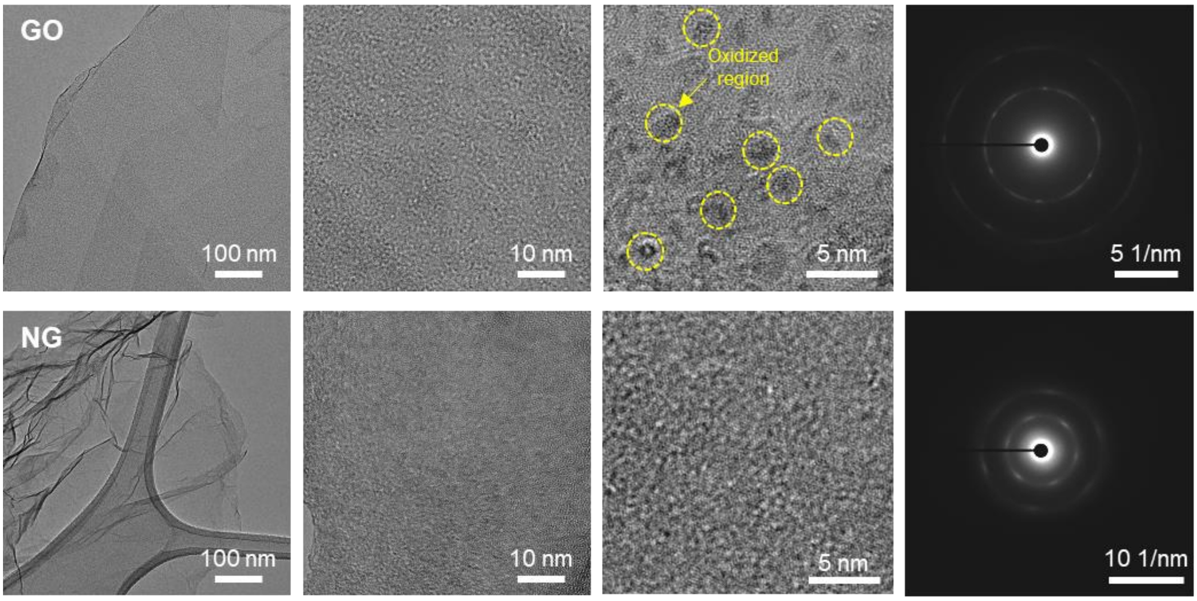

Supplementary Figure 3. HR-TEM images and SAED patterns of GO and NG. Absence of amorphous ring pattern of NG indicates that oxidized regions are removed after thermal activation. Contrastingly, the crystallinity of sp^2 regions decreased as indicated by a broad hexagonal pattern attributed to the generation of defective nanopores.

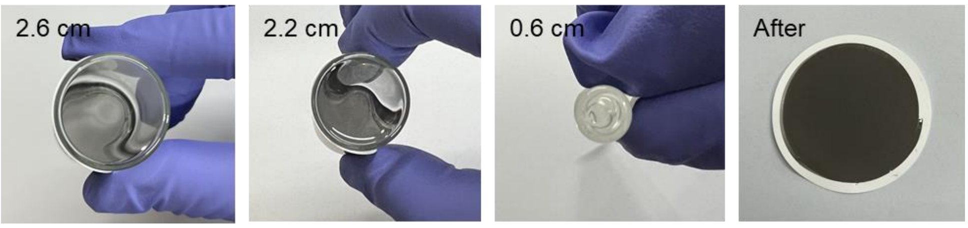

Supplementary Figure 4. Bending test of MNG membrane at several bending diameters. No cracks were observed.

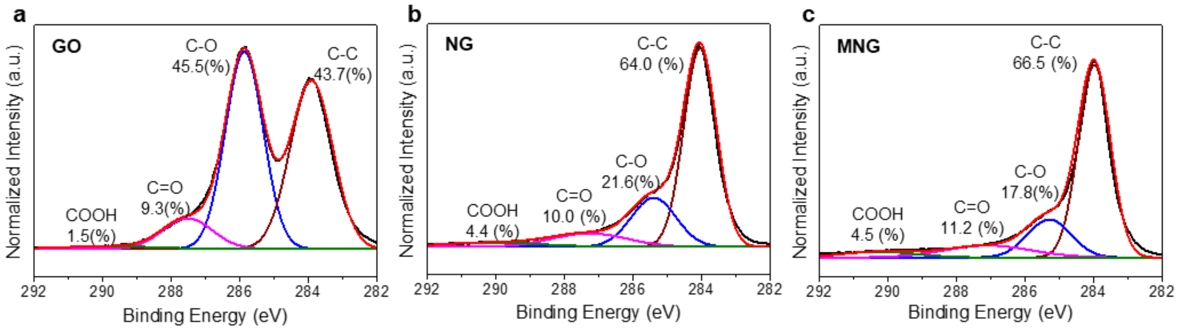

Supplementary Figure 5. a–c, XPS C1s spectra of GO, NG, and MNG.

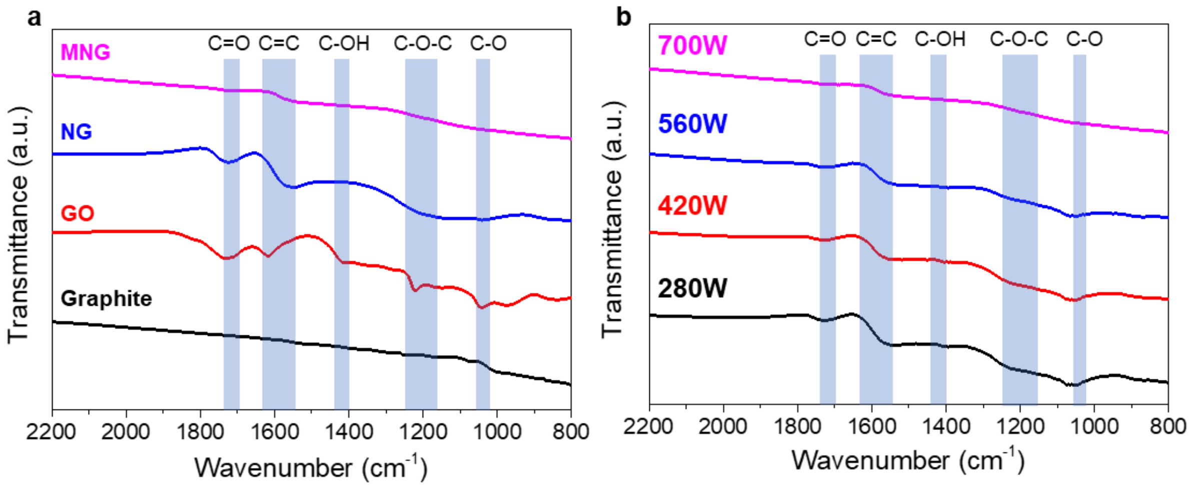

Supplementary Figure 6. a, FT-IR spectra of graphite, GO, NG, and MNG. MNG was prepared using microwave at 700 W. **b**, FT-IR of MNG depending on microwave power. All MNG powders were treated for 2 s. The peak intensities related to oxygen-containing groups successfully decreased after 700 W treatment.

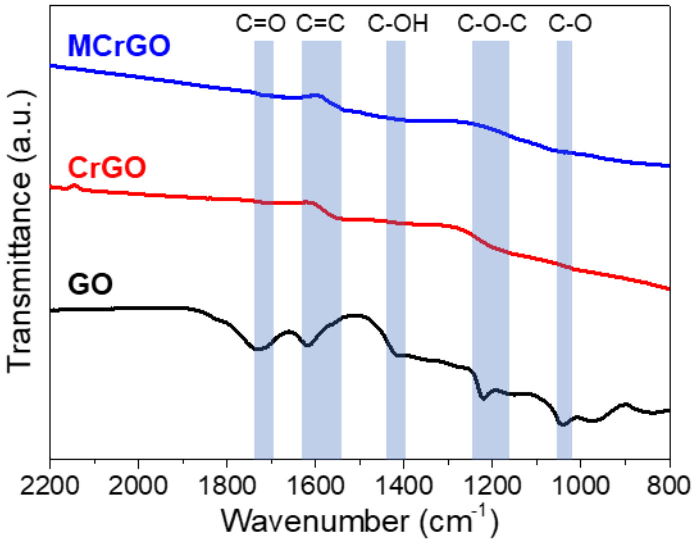

Supplementary Figure 7. FT-IR spectra of GO, CrGO, and MCrGO. CrGO was prepared by reducing GO with hydrazine hydrate. Subsequently, CrGO was treated using a 700 W microwave for 2 s to obtain MCrGO.

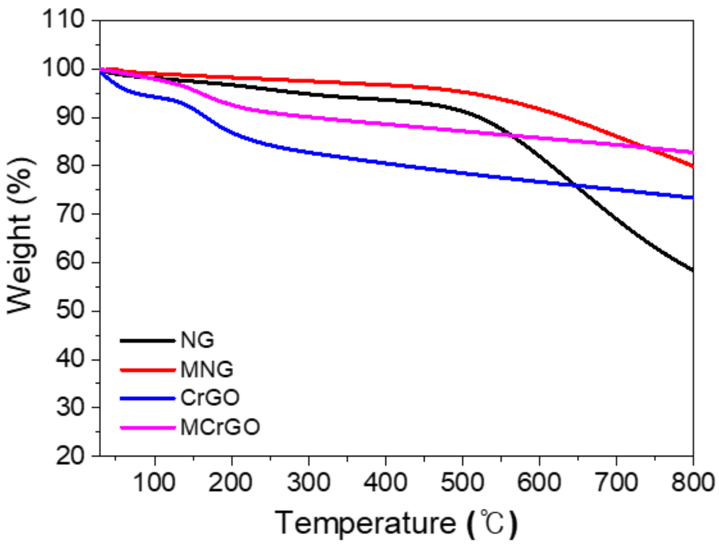

Supplementary Figure 8. TGA plots of NG, MNG, CrGO and MCrGO powders. TGA investigation was conducted in nitrogen with a heating rate of 20 °C/min. NG was prepared by thermal annealing at 200 °C for 5 min. MNG and MCrGO were treated with 700 W for 2 s on NG and CrGO, respectively.

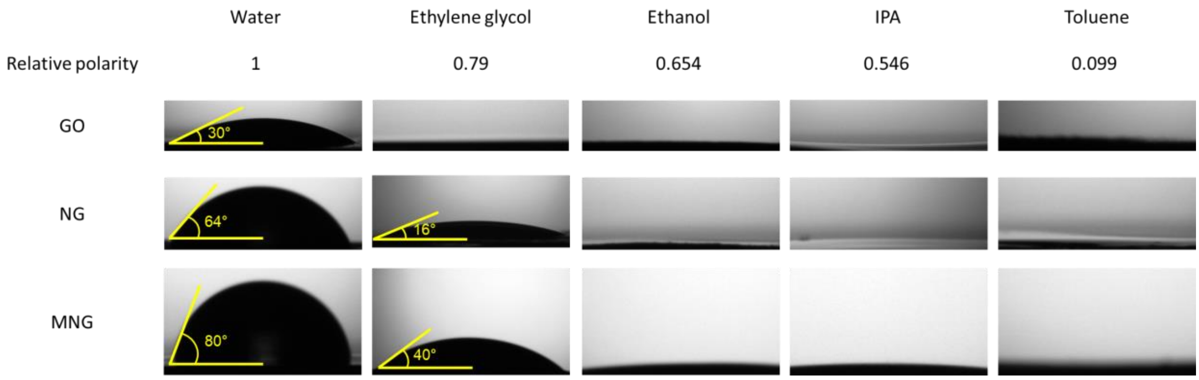

Supplementary Figure 9. The contact angles of various solvents on GO, NG, and MNG membranes.

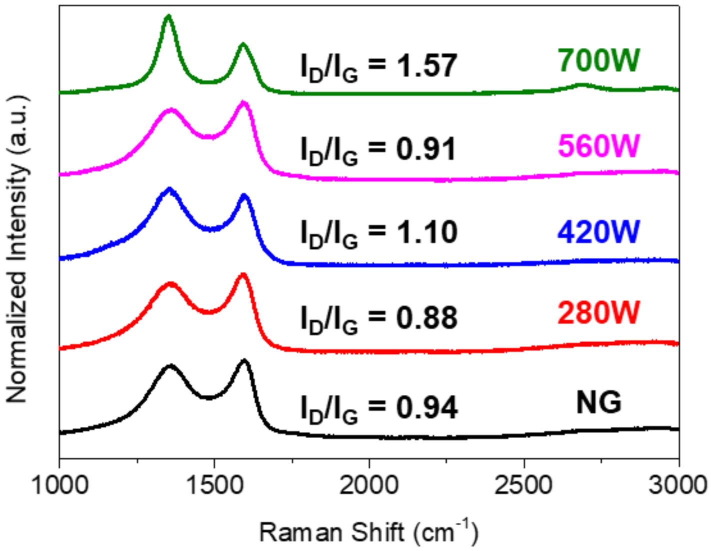

Supplementary Figure 10. Raman spectra of MNGs with different power of microwave. All MNG powders were treated for 2 s. The *D*-band intensity increased as the microwave power increased owing to the enhanced generation of defects and edge structures. The presence of *2D* peak indicates that 700 W is required to restore the sp^2 crystalline structure of NG.

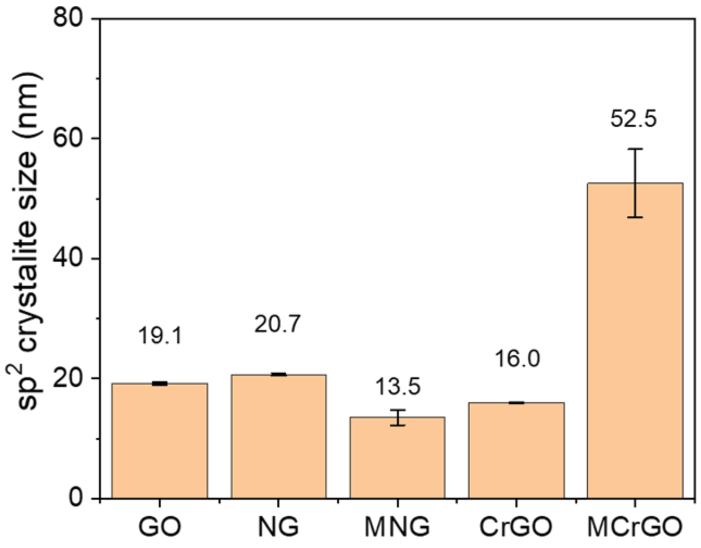

Supplementary Figure 11. Average sp^2 domain size of GO, NG, MNG, CrGO, and MCrGO. sp^2 domain size was calculated based on the Raman spectra using the following equation: $L_{sp^2} = \frac{560}{E_L^4}\frac{I_G}{I_D}$, where E_L is the energy of the Raman laser source[1].

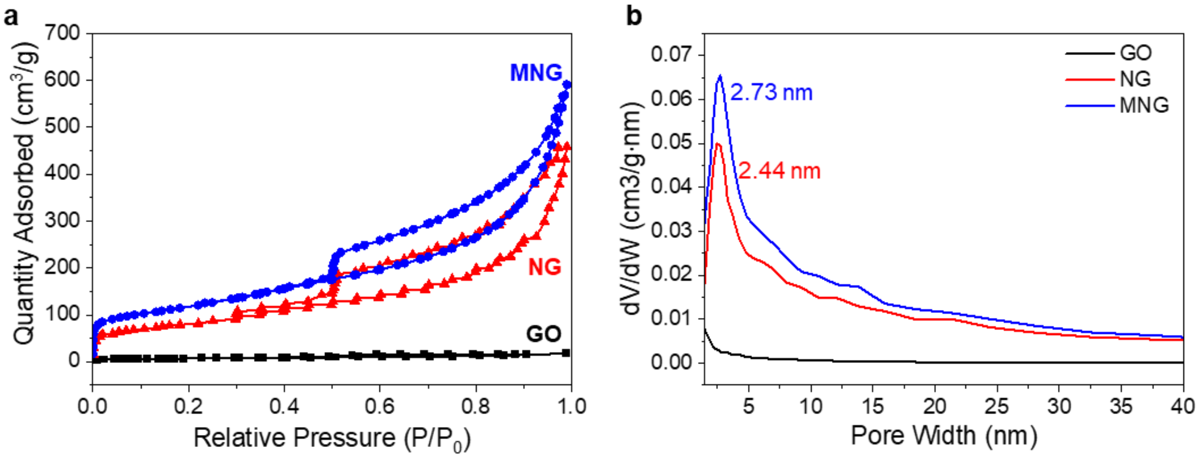

Supplementary Figure 12. **a**, N_2 adsorption–desorption isotherms of GO, NG, and MNG measured at 77 K. **b**, Pore size distribution (PSD) of GO, NG, and MNG calculated by BJH method.

Supplementary Table 1. Textural properties of prepared materials including surface areas and pore volumes.

Sample	S_{BET}[a] (m^2/g)	$S_{external}$ (m^2/g)	S_{micro}[b] (m^2/g)	V_{total}[c] (cm^3/g)	$V_{external}$ (cm^3/g)	V_{micro}[d] (cm^3/g)
GO	24.0	11.1	12.9	0.027	0.02	0.007
NG	305.8	231.2	74.6	0.74	0.62	0.12
MNG	419.1	288.1	131.0	0.91	0.74	0.17

[a] Brunauer−Emmett−Teller (BET) surface area calculated in the pressure range (P/P_0) of 0.01–0.12.

[b] Micropore surface areas calculated from the N_2 adsorption isotherms using t-plot method.

[c] Total pore volume obtained at 0.99 of P/P_0.

[d] Micropore volume calculated using t-plot method.

External pore volume and surface area are obtained by subtracting micropore volume and micropore surface area from the total values.

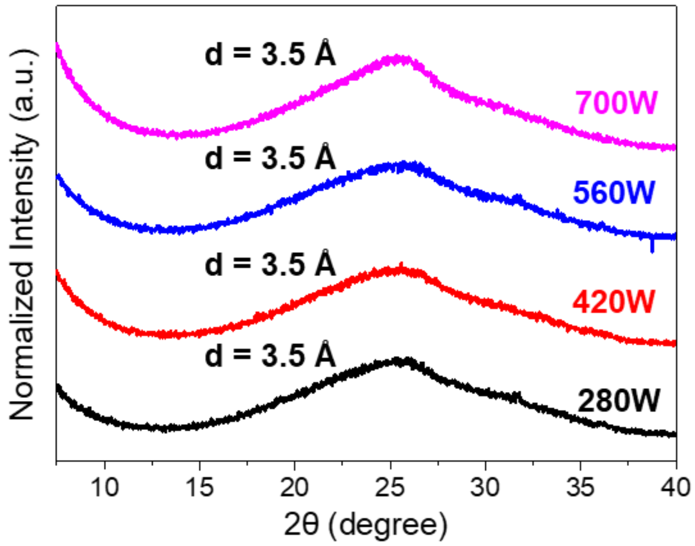

Supplementary Figure 13. XRD patterns of MNG with different microwave power. All powders were treated for 2 s. No significant difference was observed with different microwave powers on the XRD data.

Supplementary Table 2. The physical parameters of various organic solvents used in this work[2,3].

Solvent	Viscosity (mPa·s)	Kinetic diameter (nm)	Hansen solubility parameter ($MPa^{1/2}$)
Acetone	0.320	0.47	19.9
Methanol	0.549	0.41	29.6
Toluene	0.566	0.59	18.2
Water	0.935	0.27	47.8
Ethanol	1.350	0.45	26.5
NMP	1.670	0.54	23.0
IPA	2.151	0.47	24.6
Butanol	2.732	0.50	23.1

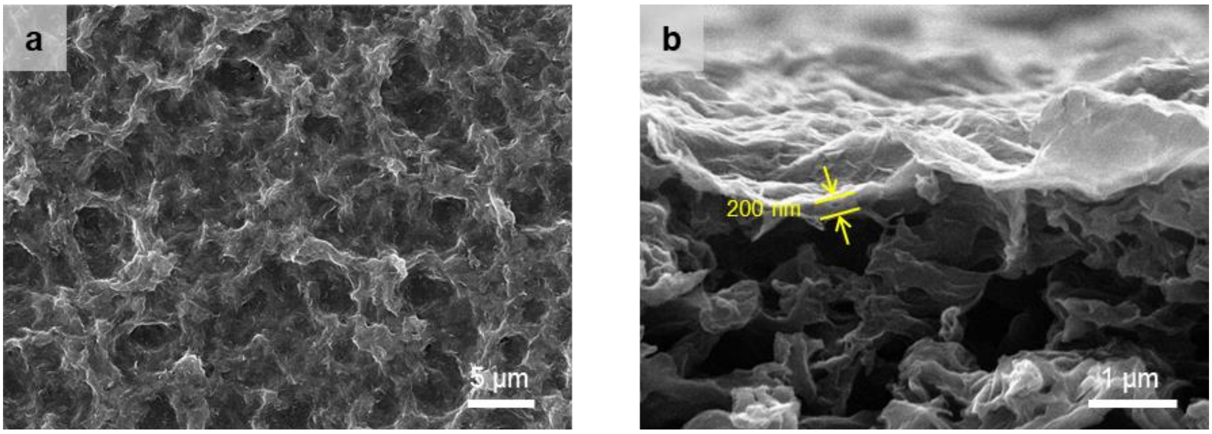

Supplementary Figure 14. SEM images of NG membranes. **a** and **b**, Top-view and cross-sectional SEM images of NG membrane on nylon support.

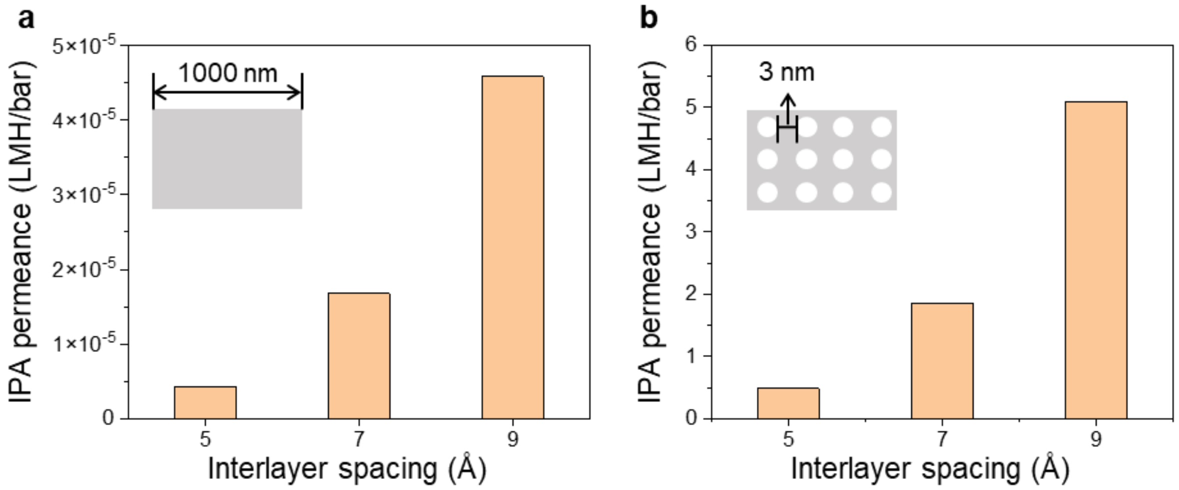

Supplementary Figure 15. Calculated IPA permeance. Here, Flux= $h^4 \Delta P/12L^2 \eta \Delta x$, where, h is the size of channel in laminates; L is the average lateral length of sheet; and Δx is membrane thickness[4]. 1000 nm (**a**) and 3 nm (**b**) of the lateral length of sheet (L) with 200 nm of Δx. The 3 nm was assumed because the distance is several nanometer size between nanopores on surface.

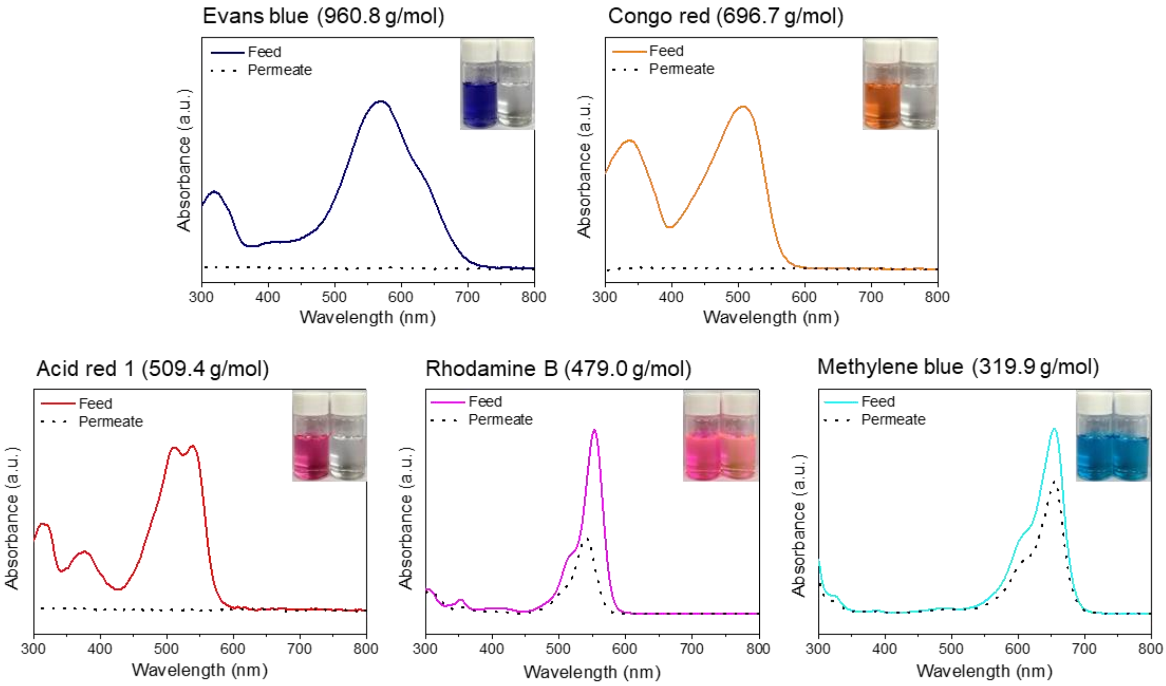

Supplementary Figure 16. UV–Visible absorbance spectra of EB, CR, AR, RhB, and MnB before and after filtration using MNG membrane. The insets depict photographs of the feed and permeate solutions. The concentration of dye solutions was 10 mg/L in IPA.

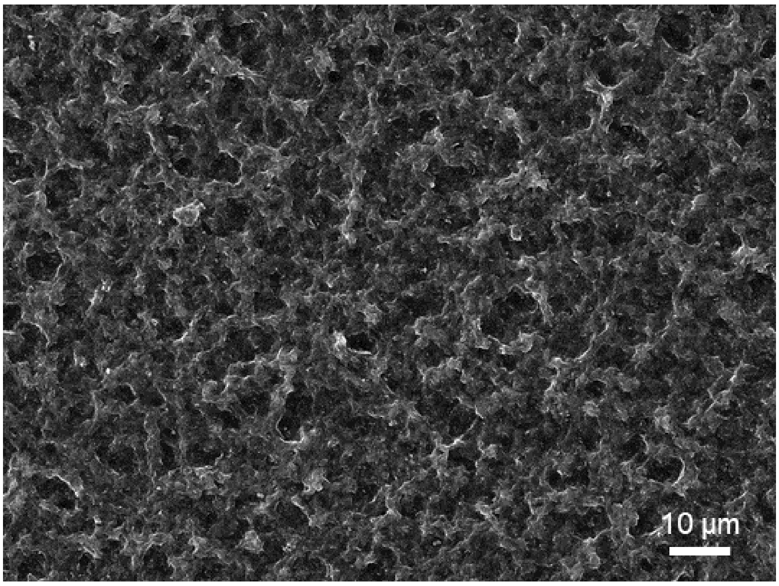

Supplementary Figure 17. SEM images of MNG membrane after EB in NMP filtration test. No defects were observed even after the filtration test.

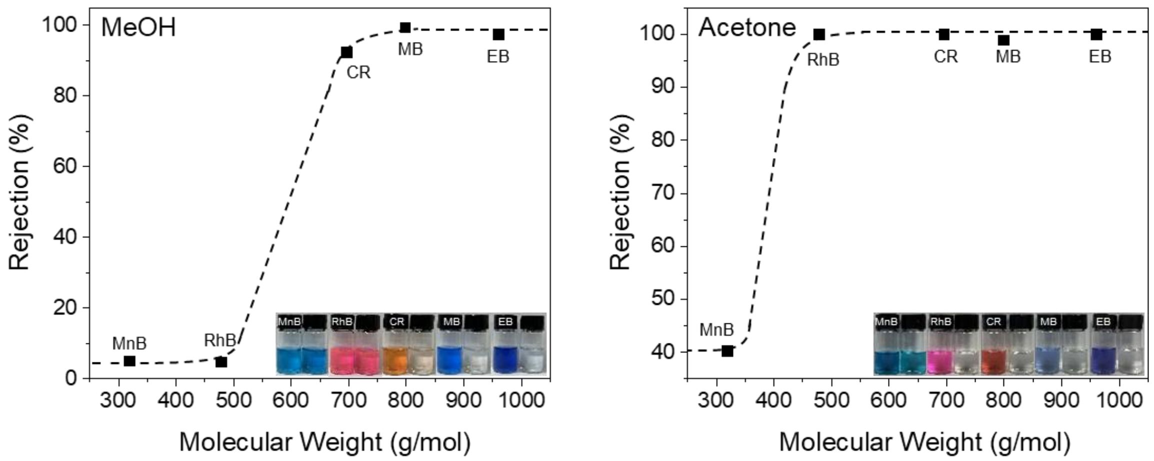

Supplementary Figure 18. OSN performance of MNG membrane in methanol and acetone.

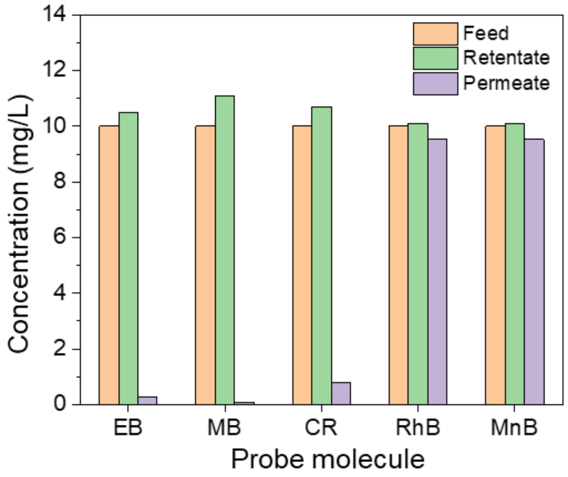

Supplementary Figure 19. Concentration of feed, retentate, and permeate solution based on the nanofiltration test result in methanol. After filtration, the retentate was concentrated because the dyes were rejected, which exhibits the rejection occurred by molecular sieving rather than adsorption.

Supplementary Table 3. A comparison of organic solvent nanofiltration performance in IPA[5–19].

Material	IPA permeance[a)]	Rejection[b)]	MWCO[c)]	Reference
PAN/PEI composited with carbon dots	3.15	PEG 1000 (99%)	709	(5)
Shear-aligned GO	130	Tannic acid (95.5%)	974	(6)
PAN/PEI-Ti3C2Tx-NH2	3	PEG800 (96%)	200	(7)
TNCS	1839	NBB (99%)	600	(8)
NH2-MWCNT/P84 MMM (M2)	1.4	Eosin Y (98.1%)	648	(9)
NG-200	241	CR (98.9%)	616	(10)
SFGO	1048	CR (95%)	697	(11)
sPPSU/0.3HPEI-WDC	3	RosB (99.9%)	600	(12)
PEG400/cPI 1:2 50W	5.91	RosB (99.6%)	748	(13)
X-PBI	5.9	Tetracycline (90.4%)	444	(14)
PAN-H/PPy IPA (4%)	3.12	RosB (97%)	974	(15)
PAN/PEI-TMC-PDMS	3.78	PEG600 (95%)	600	(16)
$(PDDA/HPE)_1$	1.8	RosB (96%)	1017	(17)
PAR-BHPF/PI	8	RosB (99%)	408	(18)
PDDA/SPEEK	1.98	AF (98%)	585	(19)
**MNG**	**2278**	**CR (99.9%) AR (99.9%)**	**509**	**This work**

[a)] Pure IPA permeance ($L\cdot m\cdot^{-2} h\cdot^{-1} bar\cdot^{-1}$).

[b)] Representative organic molecules used for filtration test.

[c)] Molecular weight cut-off: Molecular weight at 90% rejection.

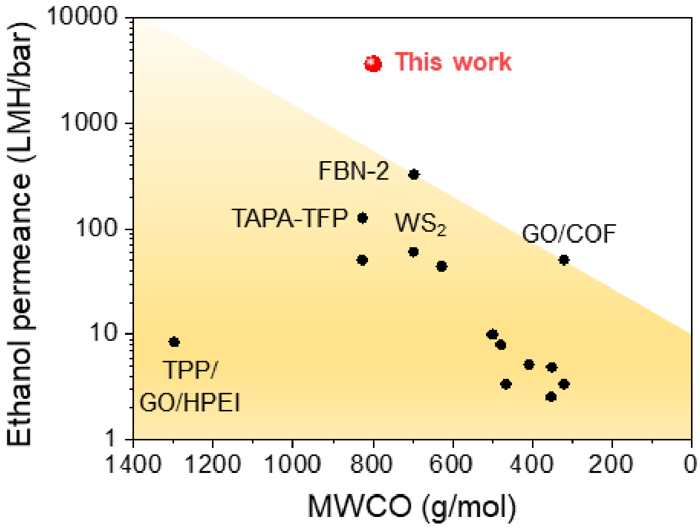

Supplementary Figure 20. Comparison of ethanol nanofiltration performances.

Supplementary Table 4. A comparison of organic solvent nanofiltration performance in ethanol[20–33].

Material	Ethanol permeance$^{a)}$	Rejection$^{b)}$	MWCO$^{c)}$	Reference
$Ti_3C_2T_x$/CNTs-CTAB	60.8	CR (>95%)	697	(20)
WS_2-NMP-15	44.4	RBB (91.5%)	627	(21)
$(3PEI/5PSS\text{-}2.5MoS_2)_{1.5}$	3.4	MnB (93.4%)	320	(22)
TAPA-TFP	127.3	BBR (94.8%)	826	(23)
GO/COF	50.8	MnB (99%)	320	(24)
FBN-2	330†	CR (>99%)	697	(25)
TPP/GO/HPEI	8.5	AB (95%)	1299	(26)
PA/cGO/cross-linked PI	4.9	RhB (99.3%)	351	(27)
Defect-sealed nanoporous graphene	50.9	RB (95.9%)	826	(28)
PA-COFs-100/PI	7.98	RhB (99.4%)	479	(29)
2%PEI2K-10	10	EY (99.9%)	500	(30)
APAN-90-3@PIM-0.10	5.2	CV (94%)	408	(31)
M_TPIM/TMC	3.4	RBB (>90%)	466	(32)
GO@PB	2.56	BS (90.2%)	352	(33)
**MNG**	**3684**	**MB (99.9%)**	**800**	**This work**

$^{a)}$ Pure ethanol permeance ($L \cdot m^{-2} \cdot h^{-1} \cdot bar^{-1}$).

$^{b)}$ Representative organic molecules used for filtration test.

$^{c)}$ Molecular weight cut-off: Molecular weight at 90% rejection.

† Dye solvent permeance

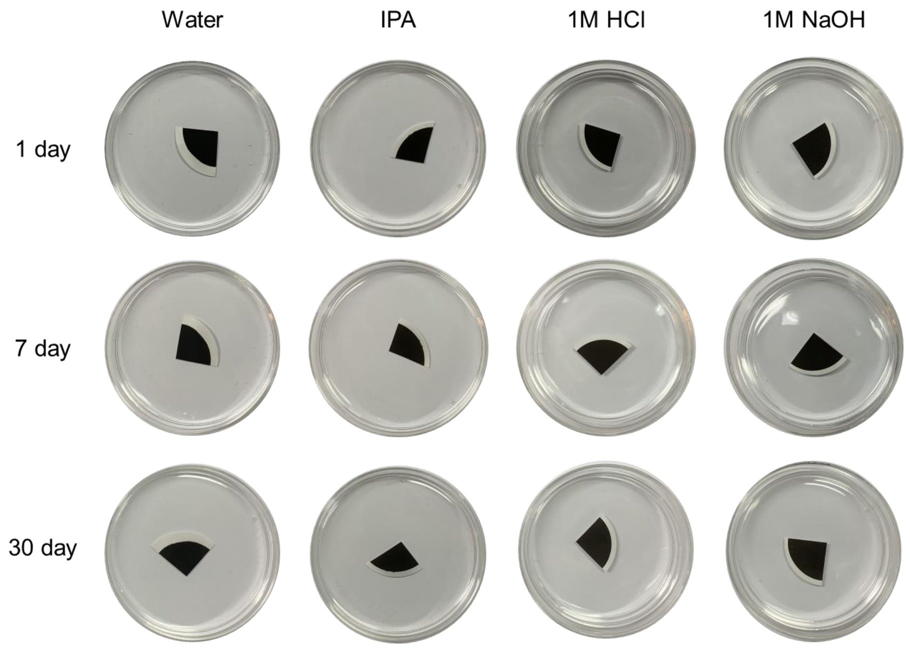

Supplementary Figure 21. Stability of MNG membrane in water, IPA, 1M HCl, and 1M NaOH for 1 d, 7 d, and 30 d, respectively. Nylon filters were used as porous supports.

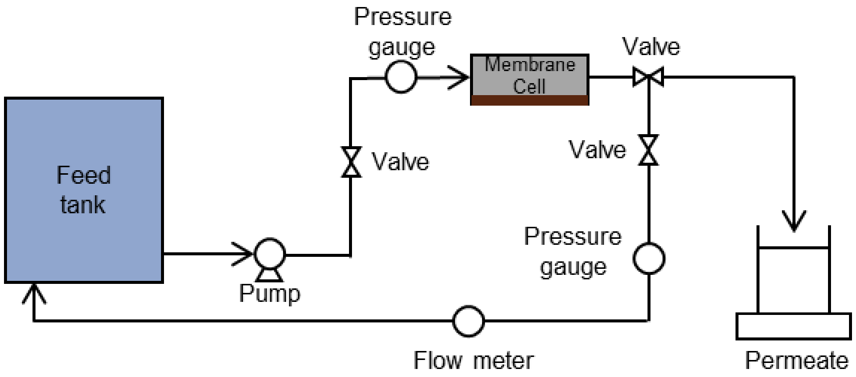

Supplementary Figure 22. Schematic illustration of the cross-filtration set-up for long-term stability test and diafiltration.

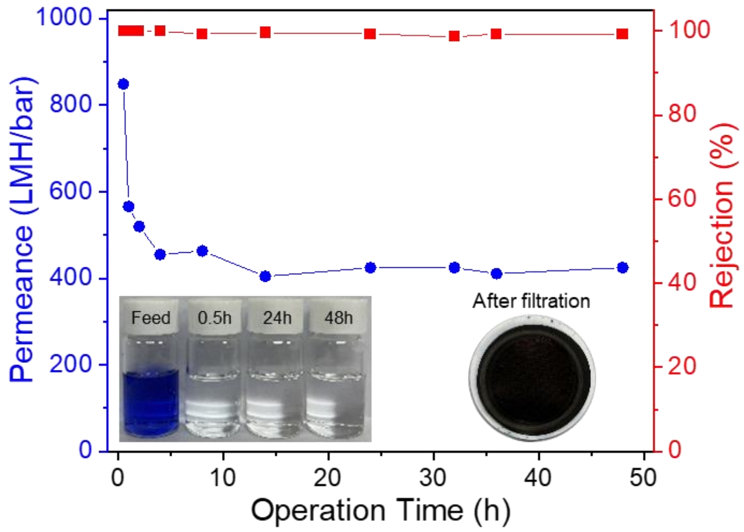

Supplementary Figure 23. Long-term filtration performance of MNG membrane under the cross-flow filtration for 2 d. The test was conducted at 2 bar with 1.7 L/min of flow rate using 10 mg/L of EB-IPA solution. The insets depict photographs of the feed and permeate solutions and MNG membrane after the cross-flow filtration test. The rejection performance was highly stable (> 99%) even after 2 d of filtration.

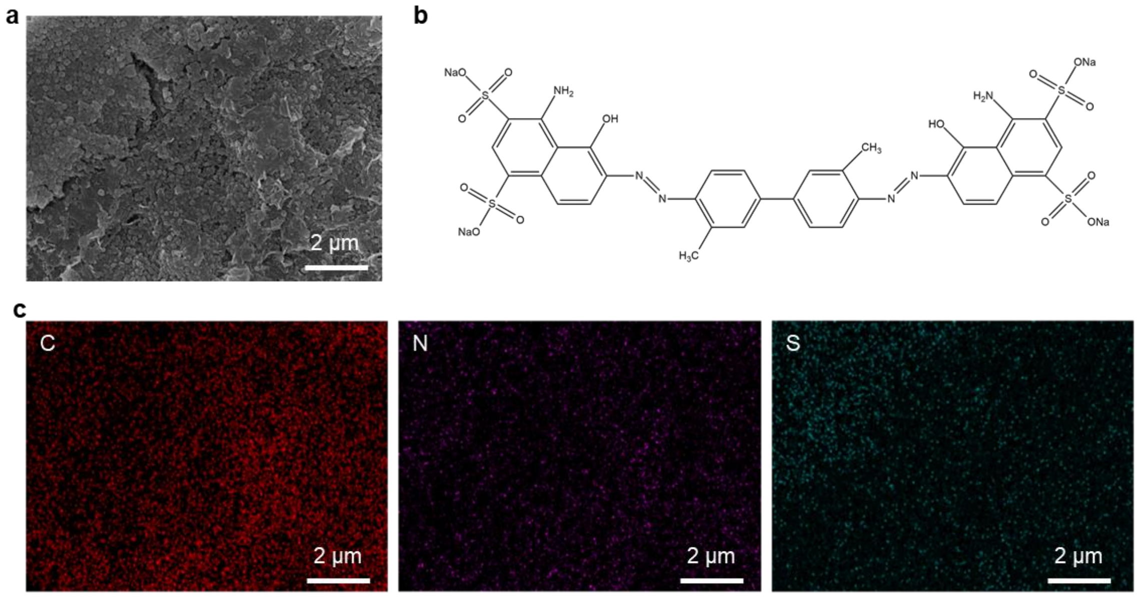

Supplementary Figure 24. MNG membrane after cross-flow filtration test with EB-IPA solution. **a**, Top-view SEM image. **b**, Chemical structure of EB molecule. **c**, EDS mapping images. Because of fast solvent permeance and low feed pressure, a thick dye cake formed.

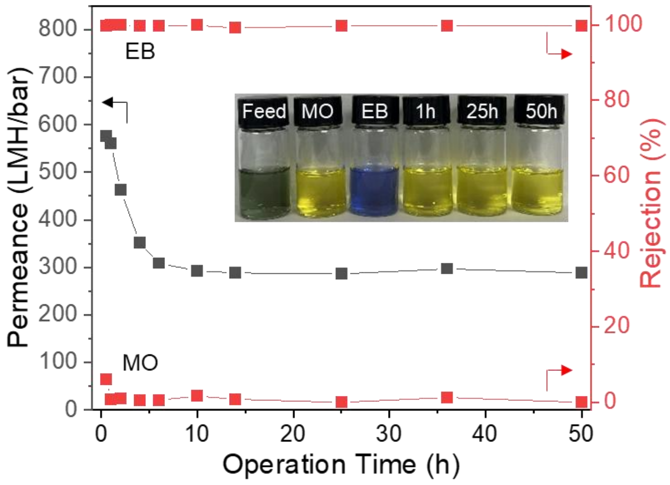

Supplementary Figure 25. Long-term diafiltration performance of MNG membrane under the cross-flow filtration for 50 h. The test was conducted at 2 bar using EB and MO in IPA at 20 mg/L of total concentration. The insets depict photographs of the mixed feed, MO, EB, and permeate solutions.

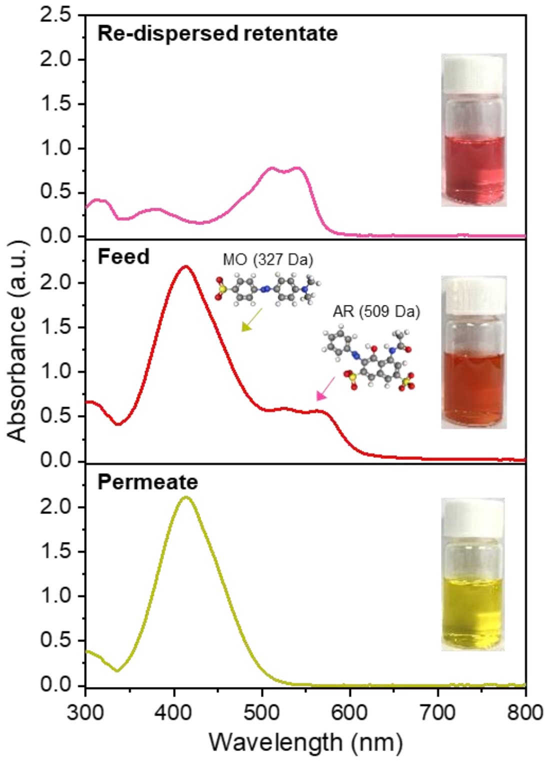

Supplementary Figure 26. MNG membrane for binary mixed dye separation under dead-end filtration. The UV–Vis absorbance spectra and photographic images of feed, permeate, and re-dispersed retentate solutions. The total concentration was 50 ppm in IPA and the weight ratio of dyes was 1:1. The test was conducted at 3 bar.

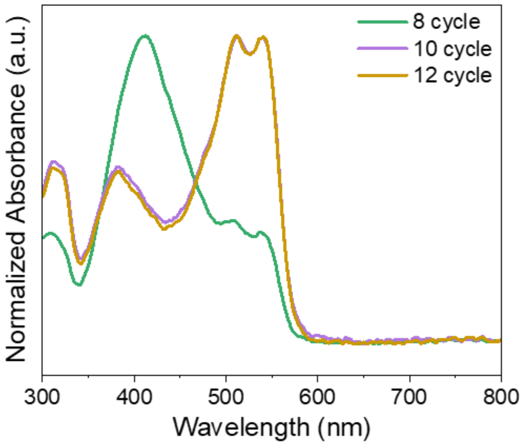

Supplementary Figure 27. UV–Visible absorbance spectra of permeate solutions after eighth filtration for the binary dye mixture filtration test. After the tenth filtration, the larger AR (509 Da) molecules were slightly permeated with almost no smaller MO (327 Da) which has already been completely extracted. The rejection rate of AR was 97% after the twelfth filtration.

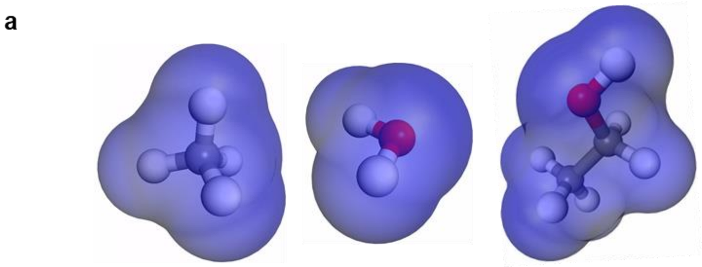

b

Solvent	Connolly Surface analysis		Diameter (Å)
Toluene (methane)	Occupied Volume(Å^3):	29.46	3.83
Water	Occupied Volume(Å^3):	20	3.37
Ethanol	Occupied Volume(Å^3):	57.69	4.79

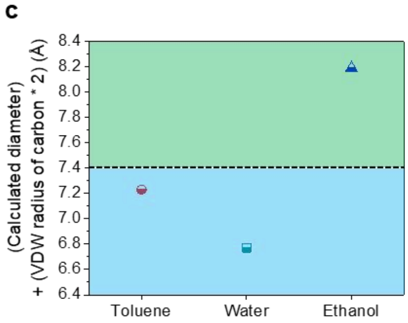

Supplementary Figure 28. a, Visualized Connolly-analyzed surfaces and volumes of toluene, water, and ethanol. For toluene, the aromatic ring is placed to be paralleled to the graphene layers, therefore its diameter was calculated using the Connolly-analyzed volume of methane. **b**, Diameters of the solvents calculated based on the Connolly-analyzed surfaces and volumes. **c**, Summarized values of solvent diameter and graphene carbon diameter for each solvent to estimate its accessible layer spacing.

Supplementary Reference

1. Cançado, L. G., *et al.* General equation for the determination of the crystallite size La of nanographite by Raman spectroscopy. *Appl. Phys. Lett.* **88**, 163106 (2006).

2. Marchetti, P., Butté, A., Livingston, A. G.. An improved phenomenological model for prediction of solvent permeation through ceramic NF and UF membranes. *J. Membr. Sci.* **415**, 444-458 (2012).

3. Nie, L., *et al.* Realizing small-flake graphene oxide membranes for ultrafast size-dependent organic solvent nanofiltration. *Sci. Adv.* **6**, eaaz9184 (2020).

4. Huang, H., *et al*. Ultrafast viscous water flow through nanostrand-channelled graphene oxide membranes. *Nat. Commun*. **4**, 2979 (2013).

5. Yuan, Z., *et al.* Carbon dots-incorporated composite membrane towards enhanced organic solvent nanofiltration performance. *J. Membr. Sci.* **549**, 1-11 (2018).

6. Akbari, A., *et al.* Solvent transport behavior of shear aligned graphene oxide membranes and implications in organic solvent nanofiltration. *ACS Appl. Mater. Interfaces* **10**, 2067-2074 (2018).

7. Hao, L., Zhang, H., Wu, X., Zhang, J., Wang, J., Li, Y. Novel thin-film nanocomposite membranes filled with multi-functional $Ti_3C_2T_x$ nanosheets for task-specific solvent transport. *Compos. Part A Appl. Sci. Manuf.* **100**, 139-149 (2017).

8. Jang, J., Nam, Y. T., Kim, D., Kim, Y.-J., Kim, D. W., Jung, H.-T. Turbostratic nanoporous carbon sheet membrane for ultrafast and selective nanofiltration in viscous green solvents. *J. Mater. Chem. A* **8**, 8292-8299 (2020).

9. Davood Abadi Farahani, M. H., Hua, D., Chung, T.-S. Cross-linked mixed matrix membranes (MMMs) consisting of amine-functionalized multi-walled carbon nanotubes and P84 polyimide for organic solvent nanofiltration (OSN) with enhanced flux. *J. Membr. Sci.* **548**, 319-331 (2018).

10. Kang, J., *et al.* Thermally-induced pore size tuning of multilayer nanoporous graphene for organic solvent nanofiltration. *J. Membr. Sci.* **637**, 119620 (2021).

11. Ruiz-Torres, C. A., *et al.* Graphene-based ultrafast nanofiltration membrane under cross-flow operation: Effect of high-flux and filtered solute on membrane performance.

Carbon **185**, 641-649 (2021).

12. Asadi Tashvigh, A., Luo, L., Chung, T.-S., Weber, M., Maletzko, C. A novel ionically cross-linked sulfonated polyphenylsulfone (sPPSU) membrane for organic solvent nanofiltration (OSN). *J. Membr. Sci.* **545**, 221-228 (2018).

13. Gao, Z. F., Shi, G. M., Cui, Y., Chung, T.-S. Organic solvent nanofiltration (OSN) membranes made from plasma grafting of polyethylene glycol on cross-linked polyimide ultrafiltration substrates. *J. Membr. Sci.* **565**, 169-178 (2018).

14. Davood Abadi Farahani, M. H., Chung, T.-S. A novel crosslinking technique towards the fabrication of high-flux polybenzimidazole (PBI) membranes for organic solvent nanofiltration (OSN). *Sep. Purif. Technol.* **209**, 182-192 (2019).

15. Li, X., Vandezande, P., Vankelecom, I. F. J. Polypyrrole modified solvent resistant nanofiltration membranes. *J. Membr. Sci.* **320**, 143-150 (2008).

16. Zhang, H., *et al.* Cross-linked polyacrylonitrile/polyethyleneimine–polydimethylsiloxane composite membrane for solvent resistant nanofiltration. Chem. Eng. Sci. 106, 157-166 (2014).

17. Joseph, N., *et al.* Ultrathin single bilayer separation membranes based on hyperbranched sulfonated poly(aryleneoxindole). *Adv. Funct. Mater.* **27**, 1605068 (2017).

18. Jimenez-Solomon, M. F., Song, Q., Jelfs, K. E., Munoz-Ibanez, M., Livingston, A. G. Polymer nanofilms with enhanced microporosity by interfacial polymerization. *Nat. Mater.* **15**, 760-767 (2016).

19. Li, X., *et al.* Solvent-resistant nanofiltration membranes based on multilayered polyelectrolyte complexes. *Chem. Mater.* **20**, 3876-3883 (2008).

20. Shao, D.-D., *et al.* Enhancing interfacial adhesion of MXene nanofiltration membranes via pillaring carbon nanotubes for pressure and solvent stable molecular sieving. *J. Membr. Sci.* **623**, 119033 (2021).

21. Tham, H. M., Japip, S., Chung, T.-S. WS_2 deposition on cross-linked polyacrylonitrile with synergistic transformation to yield organic solvent nanofiltration membranes. *J. Membr. Sci.* **588**, 117219 (2019).

22. Jiang, S.-D., Koh, A. Y. K., Chong, K. H., Zhang, S. Opening organic solvent pathways

by molybdenum disulfide in mixed matrix membranes for molecular separation. *J. Membr. Sci.* **585**, 60-66 (2019).

23. Liu, J., Han, G., Zhao, D., Lu, K., Gao, J., Chung, T.-S. Self-standing and flexible covalent organic framework (COF) membranes for molecular separation. *Sci. Adv.* **6**, eabb1110 (2020).

24. Chen, L., *et al.* High performance hierarchically nanostructured graphene oxide/covalent organic framework hybrid membranes for stable organic solvent nanofiltration. *Appl. Mater. Today* **20**, 100791 (2020).

25. Chen, C., *et al.* Functionalized boron nitride membranes with ultrafast solvent transport performance for molecular separation. *Nat. Commun.* **9**, 1902 (2018).

26. Hua, D., Chung, T.-S. Polyelectrolyte functionalized lamellar graphene oxide membranes on polypropylene support for organic solvent nanofiltration. *Carbon* **122**, 604-613 (2017).

27. Li, Y., Li, C., Li, S., Su, B., Han, L., Mandal, B. Graphene oxide (GO)-interlayered thin-film nanocomposite (TFN) membranes with high solvent resistance for organic solvent nanofiltration (OSN). *J. Mater. Chem. A* **7**, 13315-13330 (2019).

28. Cheng, C., Iyengar, S. A., Karnik, R. Molecular size-dependent subcontinuum solvent permeation and ultrafast nanofiltration across nanoporous graphene membranes. *Nat. Nanotechnol.* **16**, 989-995 (2021).

29. Li, C., Li, S., Tian, L., Zhang, J., Su, B., Hu, M. Z. Covalent organic frameworks (COFs)-incorporated thin film nanocomposite (TFN) membranes for high-flux organic solvent nanofiltration (OSN). *J. Membr. Sci.* **572**, 520-531 (2019).

30. Feng, Y., Weber, M., Maletzko, C., Chung, T.-S. Facile fabrication of sulfonated polyphenylenesulfone (sPPSU) membranes with high separation performance for organic solvent nanofiltration. *J. Membr. Sci.* **549**, 550-558 (2018).

31. Zhou, S., Zhao, Y., Zheng, J., Zhang, S. High-performance functionalized polymer of intrinsic microporosity (PIM) composite membranes with thin and stable interconnected layer for organic solvent nanofiltration. *J. Membr. Sci.* **591**, 117347 (2019).

32. Gao, J., Japip, S., Chung, T.-S. Organic solvent resistant membranes made from a cross-linked functionalized polymer with intrinsic microporosity (PIM) containing thioamide groups. *Chem. Eng. J.* **353**, 689-698 (2018).

33. Gao, Y., Su, K., Li, Z., Cheng, B. Graphene oxide hybrid poly(p-phenylene sulfide) nanofiltration membrane intercalated by bis(triethoxysilyl) ethane. *Chem. Eng. J.* **352**, 10-19 (2018).
